# Paracrine Crosstalk between Fibroblasts and ER^+^ Breast Cancer Cells Creates an IL1β-Enriched Niche that Promotes Tumor Growth

**DOI:** 10.1016/j.isci.2019.07.034

**Published:** 2019-07-24

**Authors:** Sumanta Chatterjee, Vasudeva Bhat, Alexei Berdnikov, Jiahui Liu, Guihua Zhang, Edward Buchel, Janice Safneck, Aaron J. Marshall, Leigh C. Murphy, Lynne-Marie Postovit, Afshin Raouf

**Affiliations:** 1 Department of Immunology, Faculty of Health Sciences, University of Manitoba, Winnipeg, MB R3E 0T5, Canada; 2 Research Institute of Oncology & Hematology, CancerCareManitoba, Winnipeg, MB R3E 0V9, Canada; 3 Department of Surgery, Section of Plastic Surgery, Faculty of Health Sciences, University of Manitoba, Winnipeg, MB R3A 1M5, Canada; 4 Department of Pathology, Faculty of Health Sciences, University of Manitoba, Winnipeg, MB R3E 3P5, Canada; 5 Department of Biochemistry and Medical Genetics, Faculty of Health Sciences, University of Manitoba, Winnipeg, MB R3E 0J9, Canada; 6 Department of Oncology, Faculty of Medicine and Dentistry, University of Alberta, Edmonton, AB T6G 2E1, Canada; 7 Department of Obstetrics and Gynecology, Faculty of Medicine and Dentistry, University of Alberta, Edmonton, AB T6G 2E1, Canada

**Keywords:** Molecular Mechanism of Behavior, Functional Aspects of Cell Biology, Cancer

## Abstract

Breast cancer-induced activated fibroblasts support tumor progression. However, the role of normal fibroblasts in tumor progression remains controversial. In this study, we used modified patient-derived organoid cultures and demonstrate that constitutively secreted cytokines from normal breast fibroblasts initiate a paracrine signaling mechanism with estrogen receptor-positive (ER^+^) breast cancer cells, which results in the creation of an interleukin (IL)-1β-enriched microenvironment. We found that this paracrine signaling mechanism is shared between normal and activated fibroblasts. Interestingly, we observed that in reconstructed tumor microenvironment containing autologous ER^+^ breast cancer cells, activated fibroblasts, and immune cells, tamoxifen is more effective in reducing tumor cell proliferation when this paracrine signaling is blocked. Our findings then suggest that ER^+^ tumor cells could create a growth-promoting environment without activating stromal fibroblasts and that in breast-conserving surgeries, normal fibroblasts could be a significant modulator of tumor recurrence by enhancing the proliferation of residual breast cancer cells in the tumor-adjacent breast tissue.

## Highlights

• Normal fibroblast-cancer cell interaction promotes tumor progression
• Paracrine signaling common to normal and activated fibroblasts promotes drug resistance
• Fibroblast-secreted factors create an IL1β-enriched niche for ER^+^ breast cancer cell growth

## Graphical Abstract

**Figure:**
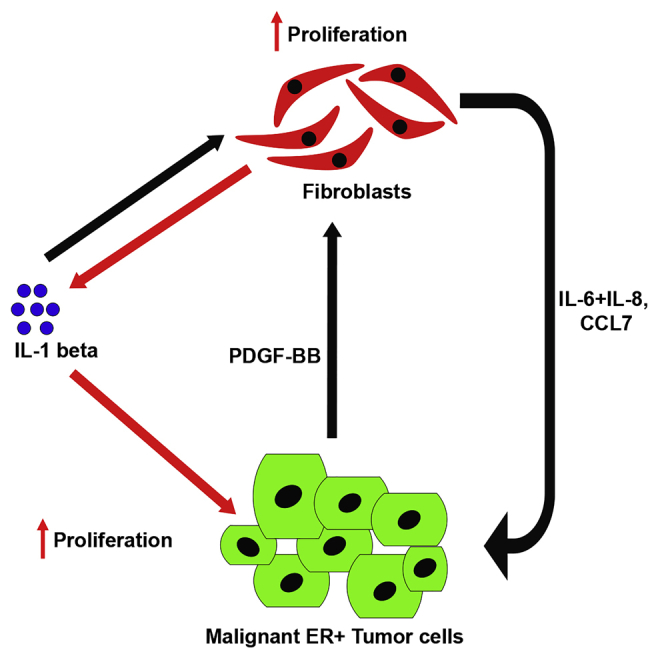

## Introduction

Current evidence suggests that cancer initiation and progression is co-mediated by the tissue environment (niche) and cancer cells rather than being a cell-autonomous-driven process (Guarnerio et al., 2018, Hanahan and Weinberg, 2011, Plaks et al., 2015). The breast cancer tumor niche consists of stromal and extracellular matrix (ECM) components, which form a complex network enabling tumor cells to maintain growth and proliferation to develop invasive and metastatic phenotypes (Acharyya et al., 2012, Lu et al., 2014, Quail and Joyce, 2013). Fibroblasts, a major stromal component of the tumor niche, have been identified as an important modifier of tumor progression (Cohen et al., 2017, Erez et al., 2010, Otomo et al., 2014). In normal breast tissue, the role of fibroblasts is to provide ECM; secrete cytokines and growth factors to support the proliferation, differentiation, and self-renewal of the epithelial stem and progenitor cells; regulate inflammatory responses; and participate in wound healing (Chatterjee et al., 2015, Chatterjee et al., 2018, Makarem et al., 2013). In the tumor niche, however, a subpopulation of fibroblasts called tumor-associated fibroblasts (TAFs) have been implicated in promoting tumor cell proliferation and cancer progression (Bussard et al., 2016, Gascard and Tlsty, 2016). These TAFs have acquired a modified and activated phenotype that is identified based on their expression of alpha-smooth muscle actin and the fibroblast activation protein (Allaoui et al., 2016, Yang et al., 2016). The activated fibroblasts make up about 40%–80% of breast tumor stroma (Bhowmick et al., 2004, Chatterjee et al., 2018, Karnoub et al., 2007, Orimo et al., 2005). Extensive studies regarding the interactions between the TAFs and breast cancer cells (BCCs) have revealed a cyclic relationship in which cancer cells stimulate a reactive response in fibroblasts and in turn activated fibroblasts enhance the proliferation and migration of cancer cells. In this regard, the secretion of transforming growth factor (TGF)-β and CXCL12 by the cancer cells has been suggested as one mechanism to confer the activated phenotype in normal stromal fibroblasts. Activated fibroblasts in turn through secretion of fibroblast growth factors, hepatocyte growth factor, interleukin (IL) 6, and TGF-β enhance cancer progression and tumor metastasis (Boimel et al., 2012, Chatterjee et al., 2018, Hugo et al., 2012, Kuperwasser et al., 2004, Pickup et al., 2013, Scherz-Shouval et al., 2014, Sharon et al., 2015).

In contrast, much less is known about the nature of interaction between BCCs and normal stromal fibroblasts. This interaction is particularly relevant in breast-conserving surgeries, wherein the reciprocal communication between tumor cells and the normal-associated fibroblasts (NAFs) could result in locoregional tumor recurrence. Initially, it was thought that NAFs prevent the development of a hyperplastic phenotype in breast epithelial cells and that they need to acquire an activated fibroblast phenotype to support breast tumor growth and progression (Dumont et al., 2013, Sadlonova et al., 2005, Shekhar et al., 2001). However, accumulating data now suggest that NAFs, similarly to TAFs, could support tumor cell proliferation (Fleming et al., 2010, Hu et al., 2008, Hu et al., 2009). This is evidenced by the recent reports indicating that addition of normal fibroblasts to patient-derived xenografts (PDXs) (Eirew et al., 2015) or MDA-MB-231 (Chatterjee et al., 2018) BCCs enhances tumor growth and take rate. These observations suggest that NAFs and TAFs may share unknown but common mechanisms to support tumor cell proliferation. In particular, the role of fibroblasts as a major component of tumor niche in supporting ER^+^ tumor cell proliferation remains unexplored due to the lack of protocols to maintain and expand primary human ER^+^BCCs *in vivo* and *ex vivo*. Recently, it was reported that primary human and mouse BCCs can be maintained in culture as organoids (Jamieson et al., 2017, Sachs et al., 2018). These assays, however, use a bulk heterogeneous population of cells, which makes the study of different subset of cells found in the tumor microenvironment difficult.

To address this gap, we developed a robust protocol to create patient-derived organoid cultures from TAFs and cancer cells separately obtained from primary malignant ER^+^ human breast tumors as well as from NAFs obtained from the normal breast tissues. Using these organoid cultures, we uncovered a pro-tumorigenic paracrine signaling mechanism common to both NAFs and TAFs. We demonstrate that the constitutively secreted CCL7 (C-C motif chemokine ligand), IL6, and IL8 from either NAFs or TAFs result in release of platelet-derived growth factor (PDGF)-BB from ER^+^BCCs, but not the normal breast cells, and that the PDGF-BB then acts in a paracrine manner causing IL1β production from NAFs and TAFs alike. IL1β then acts as a pro-proliferative factor, enhancing proliferation of both ER^+^ cancer cells and fibroblasts. Last, we show that tamoxifen (Tam) is significantly more effective as first-line therapy for malignant ER^+^ breast tumors if combined with IL1β and PDGF-BB receptor blockers.

## Results

### NAF-Secreted Factors Enhance Primary ER^+^ Breast Cancer Cell Proliferation in Organoid Cultures

**Figure 1:**
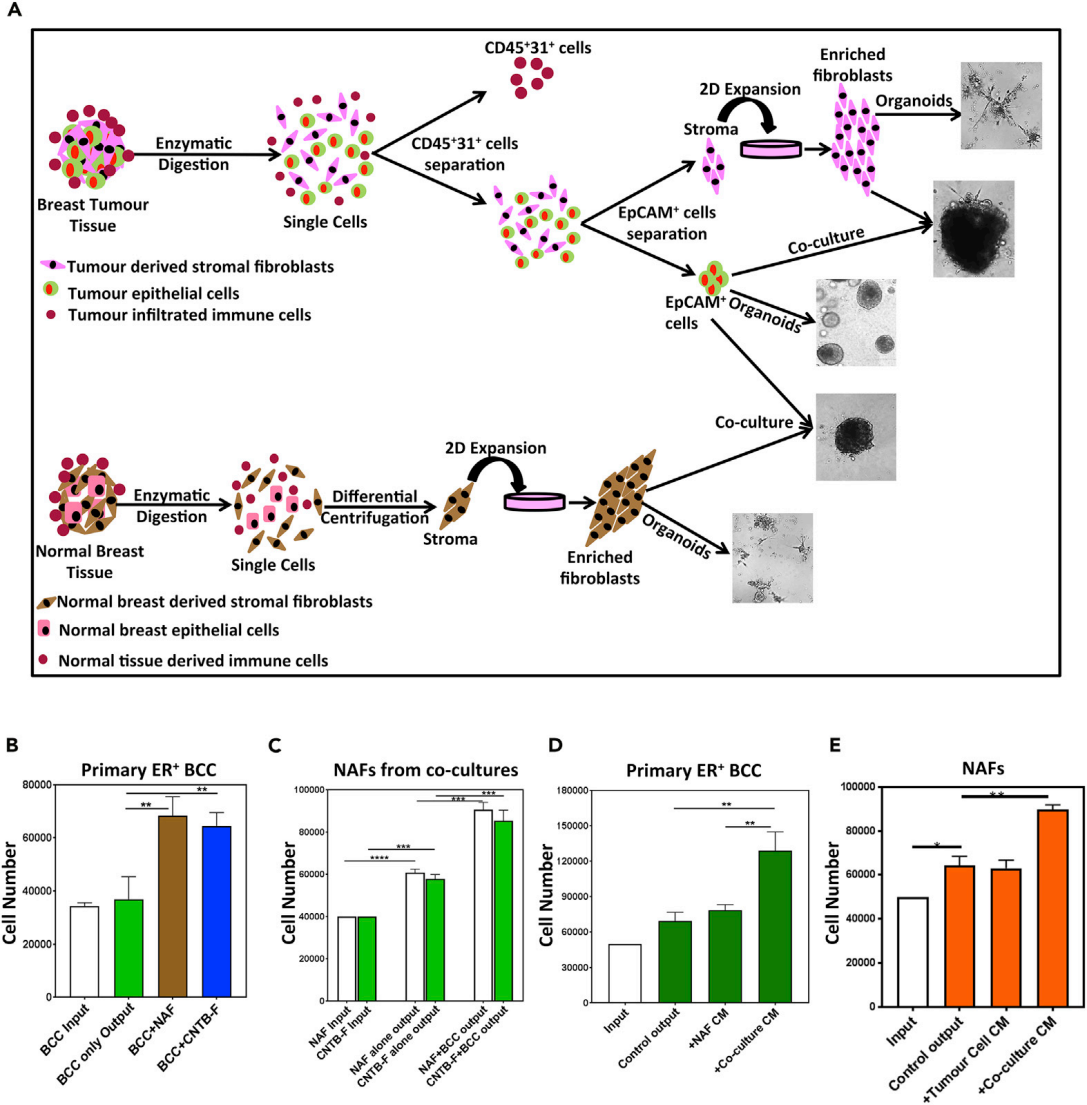
Normal Breast Fibroblasts Induce Proliferation of Primary ER^+^ Breast Cancer Cells in Organoid Cultures (A) Experimental outline of organoid co-culture system to study stromal-epithelial interactions in primary tumors. (B–E) (B) Organoid co-cultures were initiated with EpCAM^+^ primary human estrogen receptor-positive (ER^+^) breast cancer cells (BCCs) and normal breast fibroblasts obtained from either mammoplasty tissue (NAF) or matching contralateral non-tumor breast tissue (CNTB-F). Viable EpCAM^+^ BCC (B) or the EpCAM^−^ fibroblast (C) cell numbers were quantified by flow cytometry at the beginning of the cultures (input) and after 10 days (output). EpCAM^+^ BCC (D) or the EpCAM^−^ NAF (E) were placed in organoid cultures and treated with conditioned media (CM) collected from NAF and ER^+^BCC organoid co-cultures, and after 10 days total cell numbers were obtained via flow cytometry. For all the experiments, average cell numbers and standard error of the mean (SEM) are based on primary ER^+^ breast cells obtained from three individual tumors and represented as bar graphs (∗∗p < .005, ∗∗∗p < .0005, and ∗∗∗∗p < .00005).

To investigate the nature of interaction between NAFs and ER^+^BCCs, we developed a 3-dimensional (3D) organoid model system initiated with primary malignant human ER^+^ breast cancer cells (ER^+^BCCs) and either stromal fibroblast obtained from the matching original tumors or breast reduction samples for up to 10 days (Figure 1A). In these cultures, ER^+^BCCs (the EpCAM^+^ cells) maintained their low expression of basal cell markers (CK5, CK14, αSMA, and p63) but high expression of luminal cell markers (EpCAM, MUC1, CK8+18, and ERα, Figure S1A). Likewise, NAFs and TAFs maintained the expression of fibroblast markers (CD73, CD90, CD105, CD13, and FSP1, Figure S1B). Moreover, these organoid cultures allowed the maintenance of ER^+^BCCs and NAFs for up to 10 days, and for some samples increase in cell numbers was observed, although the data were variable (Figures S1C and S1D). These data suggest that our organoid culture provides a suitable *in vivo*-like environment to study the interaction between ER^+^BCCs and fibroblasts. We therefore used this organoid culture system to assess NAFs' influence on ER^+^BCC proliferation and found that only organoid cultures containing NAFs and ER^+^BCCs showed significant increase in ER^+^BCC numbers (1.97 ± 0.2-fold compared with 1.07 ± 0.24-fold, Figure 1B). Interestingly, fibroblast numbers were also increased in these organoid cultures containing NAFs and ER^+^BCCs (2.2 ± 0.08-fold compared with 1.5 ± 0.02-fold, Figure 1C). As another source of primary NAFs we used fibroblasts obtained from tumor-free breast tissue contralateral to tumor-containing breast tissue (CNTB-Fs) and obtained similar results as NAFs (Figures 1B and 1C). As well, similar data were observed in 2D co-cultures of MCF7 and T47D cells with NAFs or CNTB-Fs (Figures S1E–S1G). Together, these data indicate that reciprocal interaction between the normal fibroblasts and cancer cells can induce proliferation of the ER^+^BCCs.

To identify whether NAF-dependent proliferation of ER^+^BCCs is mediated by secreted factors, ER^+^BCCs were placed in organoid cultures for 2 days and subsequently growth medium was replaced with conditioned media (CM) obtained from NAF-ER^+^BCC organoid co-cultures or organoid cultures containing each cell type separately for an additional 8 days when BCC (EpCAM^+^) and NAF (EpCAM^−^) cell numbers were analyzed (Figures 1D and 1E). Interestingly, only CM from the NAF-ER^+^BCC organoid co-cultures significantly increased the proliferation of BCCs (2.58 ± 0.3-fold compared with 1.38 ± 0.14-fold) and NAFs (1.87 ± 0.2 fold-compared with 1.22 ± 0.08-fold) compared with the other CMs (Figures 1D and 1E). These data suggest that interaction of NAFs with ER^+^BCCs results in secretion of pro-proliferative factors, which are not present in the ER^+^BCCs or NAF-only cultures, and that these secreted factors are sufficient to enhance the proliferation of both cell types in these cultures without the requirement for cell-cell contact.

### IL1β in NAF-BCC Co-culture CM Induces ER^+^BCCs Proliferation

**Figure 2:**
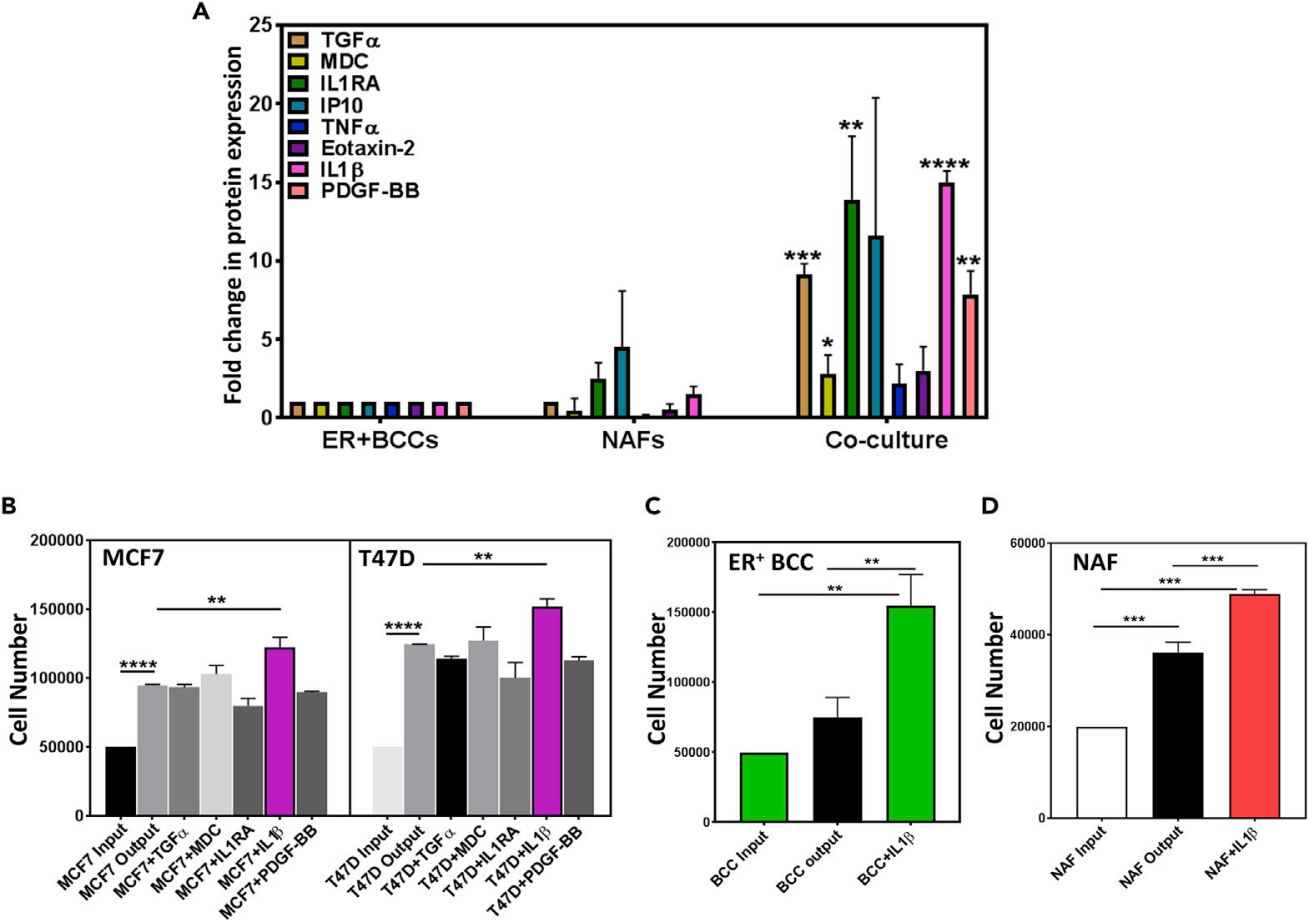
Co-culturing ER^+^BCCs with NAFs Results in IL1β Secretion that Induces Proliferation of both Cell Types (A) Cytokine ELISA array analysis of conditioned media (CM) obtained from organoid cultures consisting of EpCAM^+^ ER^+^BCC only, NAF only, or co-cultures of both cell types identified five cytokines to be significantly upregulated in the co-cultures (Table S2). Average from three biological replicates and standard error of the mean (SEM) are plotted as bar graphs where average cytokine levels in BCCs are set to 1. (B) MCF7 and T47D cells were placed in organoid cultures and treated with different cytokines for 8 days, and average cell numbers and SEM from three independent experiments are depicted in the bar graphs. (C and D) (C) ER^+^BCCs and (D) NAFs were grown separately as organoids in the presence of recombinant IL1β (rIL1β) for 8 days. Average cell numbers and SEM are based on primary ER^+^ breast cells obtained from three individual tumors and plotted as bar graphs. (∗p < .05, ∗∗p < .005, ∗∗∗p < .0005, and ∗∗∗∗p < .00005).

Secreted cytokines and growth factors present in the CM collected from organoid cultures containing both NAF and ER^+^BCCs or single cultures of each cell type were analyzed using a cytokine ELISA array (Figure 2A and Table S1). Eight cytokines were present at significantly higher levels in the CM of NAF-ER^+^BCC co-cultures compared with the CM of NAF- or BCC-only cultures (Figure 2A). Of these eight, five cytokines (TGF-α, MDC, IL1RA, IL1β, and PDGF-BB) were selected for further examination based on their significant differential secretion (>3- to 5-fold). Interestingly, only recombinant IL1β (rIL1β) significantly increased the proliferation of MCF7 and T47D BCCs (Figure 2B) and the primary ER^+^BCCs (Figure 2C), and NAFs (Figure 2D) grown in organoid cultures compared with the vehicle controls. Although rIL1β induced proliferation of both the cancer cells and fibroblasts, *IL1β* target genes were significantly upregulated in the NAFs (Figure S2A), but not in MCF7 (Figure S2B).

In contrast to the pro-proliferative effect of rIL1β on ER^+^BCCs and NAFs, rIL1β showed an antiproliferative effect on normal breast epithelial progenitor cells. Recombinant IL1β significantly impaired acinar structure formation by normal breast epithelial cells in Matrigel (Figure S2C), decreased CD49f and EpCAM progenitor marker expression (2.1 ± 0.3-fold and 1.64 ± 0.2-fold respectively, Figure S2D), decreased total cell number in Matrigel (3.33 ± 0.64-fold, Figure S2E), and significantly decreased the progenitor cell proliferation (1.73 ± 0.25-fold, Figure S2F).

### IL1β Is Secreted by Fibroblasts and Not the Breast Cancer Cells in NAF-BCC Co-cultures

**Figure 3:**
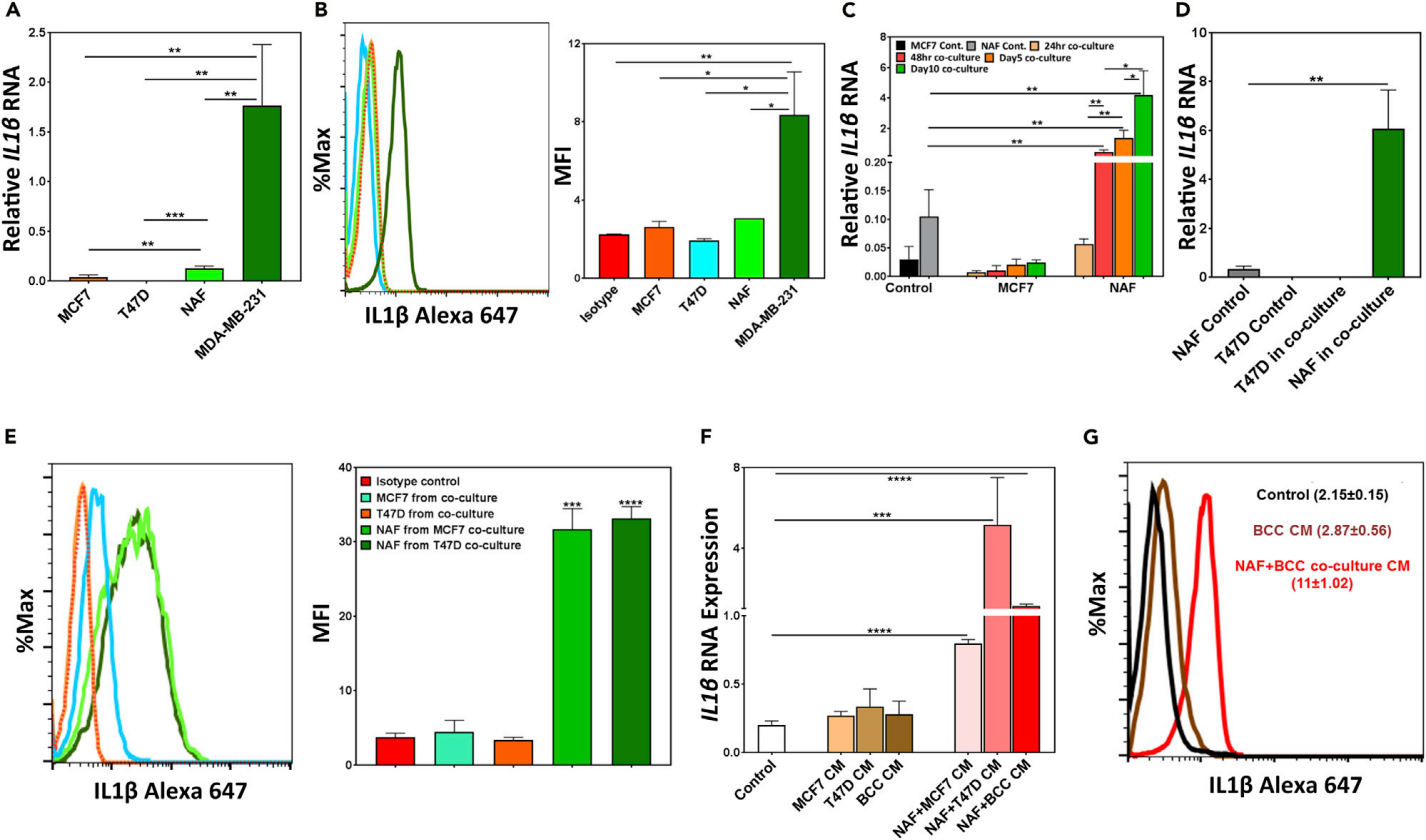
Fibroblasts Produce IL1β in Organoid Co-cultures with ER^+^ Breast Cancer Cells (A–D) *IL1β* transcripts (A) and protein (B) levels were measured in MCF7, T47D, NAFs, and MDA-MB-231 cells by qPCR and intracellular flow cytometry. NAFs were co-cultured with MCF7 (C) and T47D (D) cells and after respective days, breast cancer cells (EpCAM^+^) and NAFs (EpCAM^−^) were separated by flow cytometry and *IL1β* transcript expression was obtained by qPCR. (E) IL1β protein expression was measured in NAFs co-cultured with MCF7 and T47D by intracellular flow cytometry. (F) NAFs were treated with conditioned media (CM) obtained from either breast cancer cell (MCF7, T47D, and primary ER^+^BCC)-only cultures or from NAFs and breast cancer cells in co-cultures for 6 h, and *IL1β* transcript expression was measured by qPCR. (G) IL1β protein expression was measured in NAFs by intracellular flow cytometry following treatment with CM from ER^+^BCC-only cultures or NAF and ER^+^BCC co-cultures. All the data are represented as either bar graphs or bold text with mean ± SEM and are based on primary ER^+^ breast cells obtained from three individual tumors (∗p < .05, ∗∗p < .005, ∗∗∗p < .0005, and ∗∗∗∗p < .00005).

To understand the source of IL1β in the organoid cultures, we examined IL1β expression in the co-cultures of ER^+^ MCF7 and T47D cells with NAFs. MCF7, T47D, and NAFs express very low levels of *IL1β* transcripts and protein compared with the triple-negative MBA-MD-231 cells (Figures 3A and 3B). To ascertain the contribution of each cell type in IL1β production, MCF7 and T47D cells were placed in 2D adherent co-cultures with NAFs for up to 10 days. The EpCAM^+^ MCF7 and T47D cells were separated from the EpCAM^−^ NAFs in these co-cultures using flow cytometry and *IL1β* transcripts, and protein levels were quantified. In co-cultures, high levels of *IL1β* transcripts and proteins were detected only in the NAFs, but not in the BCCs (Figures 3C–3E), suggesting that the presence of ER^+^BCCs induces IL1β production in NAFs. To assess if secreted factors are responsible for the BCC-induced IL1β secretion from fibroblasts, NAFs were treated with CM obtained from cultures initiated with MCF7, T47D, or primary ER^+^BCCs only or in co-culture with NAFs, and *IL1β* transcript levels and protein expressions were examined. Interestingly, only the CM from the co-cultures, but not the BCC-only cultures, was able to induce *IL1β* transcript (after 6-h exposure to CM) and protein levels in NAFs (Figures 3F and 3G). This is in keeping with our observation that rIL1β enhanced *IL1β*-target genes' expression after 6 h (Figure S2A).

To examine, whether different sources of fibroblasts can also secrete IL1β in co-cultures through similar mechanisms, MCF7 and T47D cells were placed in co-cultures with fibroblasts obtained from primary ER^+^ human breast tumor adjacent tissue (TAT-Fs), CNTB-Fs, and NAFs. Fibroblasts isolated from different sources showed low *IL1β* transcript levels as in NAFs (Figure S3A). However, when placed in co-cultures with BCCs (Figure S3B) or exposed to the CM from these co-cultures (Figure S3C), *IL1β* transcript expression was significantly increased in all fibroblasts regardless of their origin. Interestingly, TAT-Fs showed much higher expression of *IL1β* transcripts and induced proliferation of T47D cells when placed in co-cultures or exposed to CM from T47D co-cultures compared with NAFs and CNTB-Fs (Figures S3B–S3E).

### Fibroblast-Induced PDGF-BB Secretion by Breast Cancer Cells Causes IL1β Secretion from Fibroblasts

**Figure 4:**
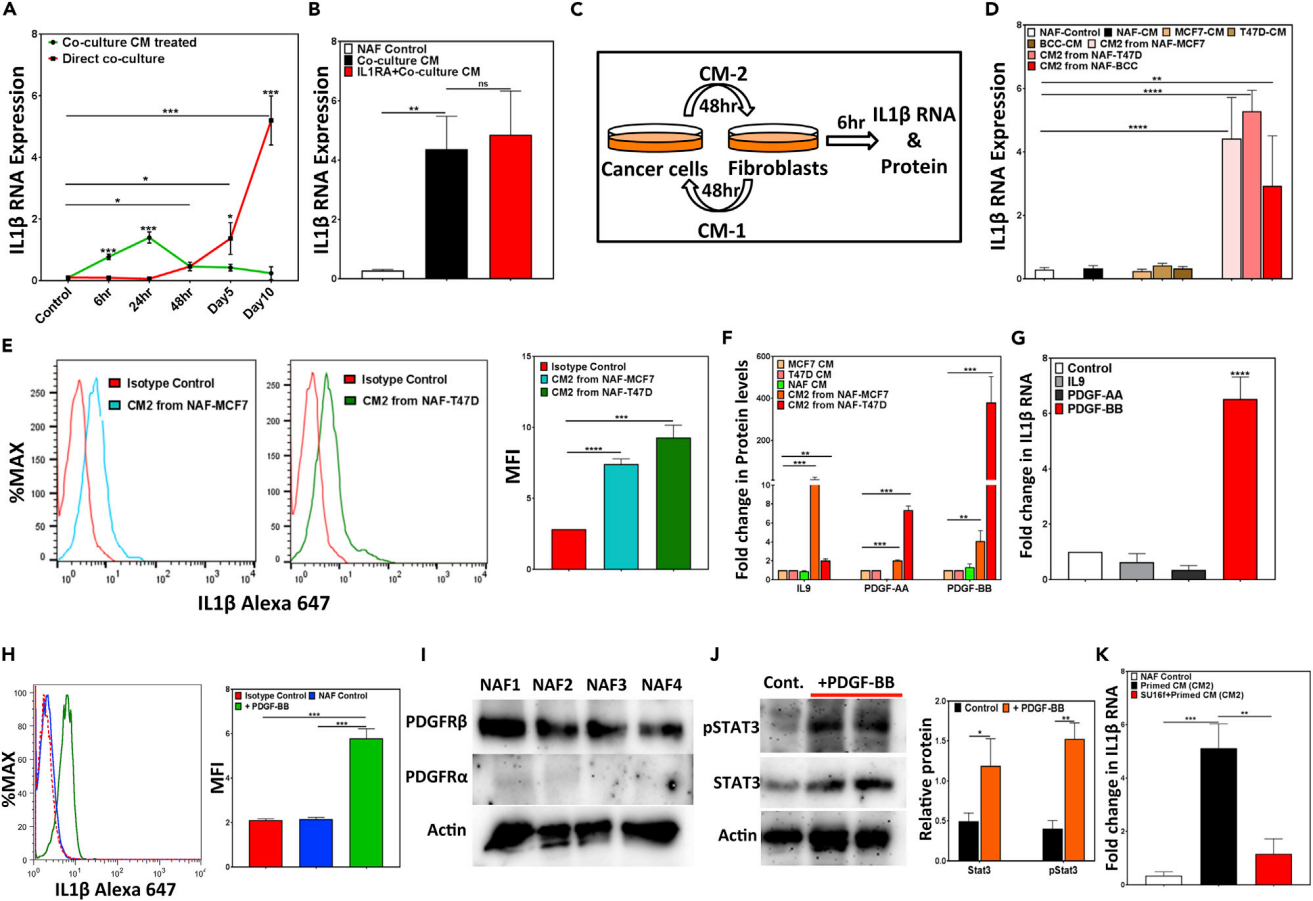
ER^+^BCC-Induced IL1β Production from Fibroblasts Requires Secreted Factors from Fibroblasts (A) *IL1β* transcript levels were quantified in NAFs either in co-cultures with MCF7 or treated with co-cultured conditioned media (CM) for up to 10 days. (B) NAFs were treated with either IL1RA or vehicle controls for 48 h and then with NAF and MCF7 co-culture CM for 6 h and *IL1β* transcript level was measured. (C) Experimental outline of 2D cultures for (D–F). (D) *IL1β* transcript expression was measured in NAFs following treatment with CM1 and CM2 for 6 h. (E) IL1β protein expression was measured in NAFs by intracellular flow cytometry following exposure to CM2 for 48 h. (F) Cytokine ELISA array was used to compare cytokine levels in CMs from NAFs, MCF7, T47D, and CM2 (also Table S2). Cytokine expression is represented as fold changes. (G) *IL1β* transcript levels were quantified in NAFs following 6 h treatment with recombinant IL9, PDGF-AA, and PDGF-BB. (H) IL1β protein levels were quantified in NAFs following 48 h treatment with recombinant PDGF-BB. (I) Representative western blots showing PDGFRα and PDGFRβ expressions in four different NAF samples. (J) Representative western blot showing total STAT3 and phospho-STAT3 protein expression in NAFs treated with PDGF-BB. Average pSTAT3 expression is normalized to total STAT3 expression and shown as bar graphs. (K) IL1β transcript expression was measured in NAFs following treatment with CM2 for 6 h with or without 48-h pre-incubation with SU16f. All data are represented as bar graphs with mean ± SEM from three independent experiments (∗p < .05, ∗∗p < .005, ∗∗∗p < .0005, and ∗∗∗∗p < .00005).

To understand the dynamics of IL1β production by NAFs, we examined *IL1β* transcript levels in NAFs either in co-culture with BCCs (MCF7) or treated with co-culture CM for up to 10 days. Interestingly *IL1β* transcript levels in NAFs treated with co-culture CM increased as early as 6 h (6.99 ± 1.7-fold) and continued to increase up to 24 h (12.5 ± 1.5-fold), but sharply declined thereafter (Figure 4A). In the co-cultures, however, *IL1β* transcript levels in NAFs significantly increased only after 48 h and continued to rise thereafter (Figure 4A). As MCF7, T47D, and NAFs express IL1β receptor IL1R1 ubiquitously (Figure S4A), it is possible that IL1β expression is regulated through a feedforward mechanism. However, blocking IL1β receptor IL1R1 with a receptor antagonist (IL1RA) could not prevent the co-culture CM-induced *IL1β* transcript increase in NAFs (Figure 4B), suggesting that secreted factors other than IL1β are responsible for increased IL1β production in NAFs. To separately assess the contribution of secreted factors from the NAFs and the BCCs to the elevated IL1β levels, MCF7, T47D, or primary ER^+^BCCs were exposed to CM obtained from NAF-only cultures for 48 h to obtain “primed-CM” (CM2, Figure 4C) and *IL1β* transcript levels were examined in NAFs exposed to the primed-CM for 6 h (Figure 4D). As controls, NAFs were also exposed to CM from MCF7, T47D, or primary ER^+^BCCs-only cultures. Interestingly, only primed-CM significantly increased *IL1β* transcripts and protein levels in NAFs (Figures 4D and 4E), suggesting that secreted factors from both NAFs and BCCs are required to cause IL1β production from NAFs.

To identify secreted cytokines and growth factors involved in the crosstalk between NAFs and BCCs, CM obtained from NAF, MCF7, and T47D as well as the “primed-CM” (CM2) were analyzed using cytokine ELISA arrays. Three cytokines (IL9, PDGF-AA, and PDGF-BB) were identified to be significantly elevated in CM2 compared with the NAF- or BCC-only CMs (Figure 4F) of which, only recombinant PDGF-BB induced *IL1β* transcript (6.5 ± 0.79 fold) and protein (2.68 ± 0.11 fold) expression in NAFs after 6 and 48 h, respectively (Figures 4G and 4H, Table S2). It is noteworthy that NAF-T47D co-culture CM contained the highest PGDF-BB level (Figure 4F). Mechanistically, PDGF-BB signaling is conducted through interaction with its cognate cell surface receptors and activation of *STAT* signaling. Interestingly, NAFs show strong expression for the β- but not the α-form of the PDGF-BB receptor (Figure 4I) and that PDGF-BB activates *STAT3* signaling in these cells (Figure 4J). Moreover, activation of PDGF receptors is required for primed-CM-induced IL1β production from NAFs (Figure 4K), because the response was eliminated by pre-incubation with the selective PDGF-BB receptor inhibitor SU16f. In contrast to ER^+^BCCs, the IL1β and PDGF-BB levels did not increase in 3D co-cultures of normal EpCAM+ primary breast epithelial cells and NAFs (Figures S4B–S4D). Also, EpCAM+ primary breast epithelial cells treated with recombinant PDGF-BB for 6 h did not increase IL1β transcript expression in them (Figure S4E). However, similar to IL1β, recombinant PDGF-BB also induced proliferation of NAFs in cultures (Figures S4F–S4H).

### Constitutively Secreted Cytokines from NAFs Induce PDGF-BB Secretion from Breast Cancer Cells

**Figure 5:**
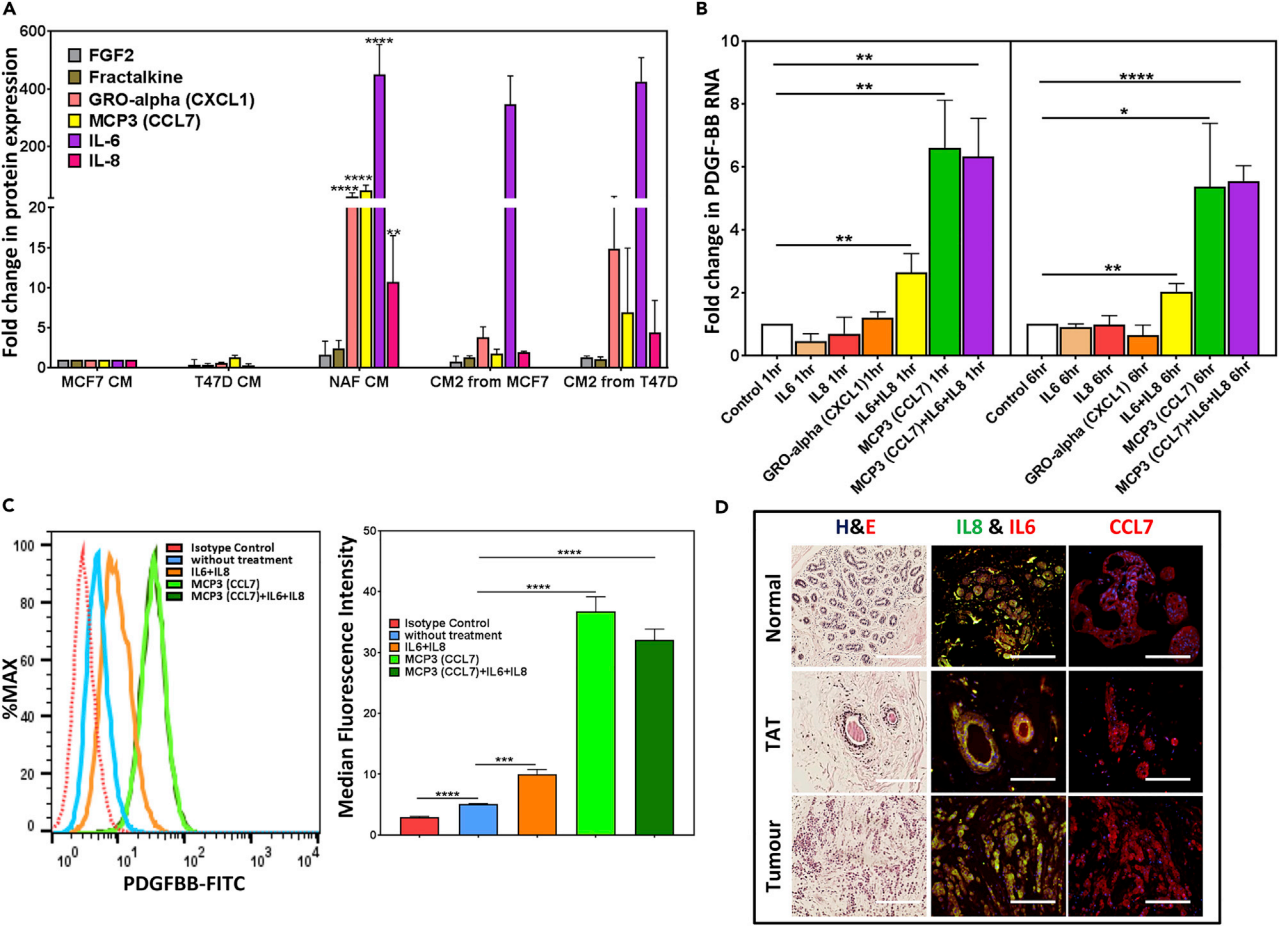
NAF-Secreted CCL7, IL6, and IL8 Induce PDGF-BB Production from Primary ER^+^BCCs (A) Cytokine array data (Figure 4F) identified CCL7, IL6, IL8, and GROα as cytokines highly secreted by only NAFs, but not by the MCF7 and T47D cells. (B) *PDGFB* transcript levels were quantified via qPCR in EpCAM^+^ primary ER^+^BCC after 1- and 6-h treatment with recombinant IL6, IL8, CCL7, and GROα either alone or in combination. (C) PDGF-BB protein expression in primary ER^+^BCCs was quantified after 48-h treatment with recombinant CCL7, IL6, and IL8. All data are represented as bar graphs with mean ± SEM from three independent experiments (∗p < .05, ∗∗p < .005, ∗∗∗p < .0005, and ∗∗∗∗p < .00005). (D) Representative H&E and immunofluorescent images from three independent experiments showing IL6, IL8, and CCL7 protein expression in ER^+^ breast tumor, matched tumor-adjacent, and healthy normal breast tissue sections. Scale bars, 200 μm.

**Figure 6:**
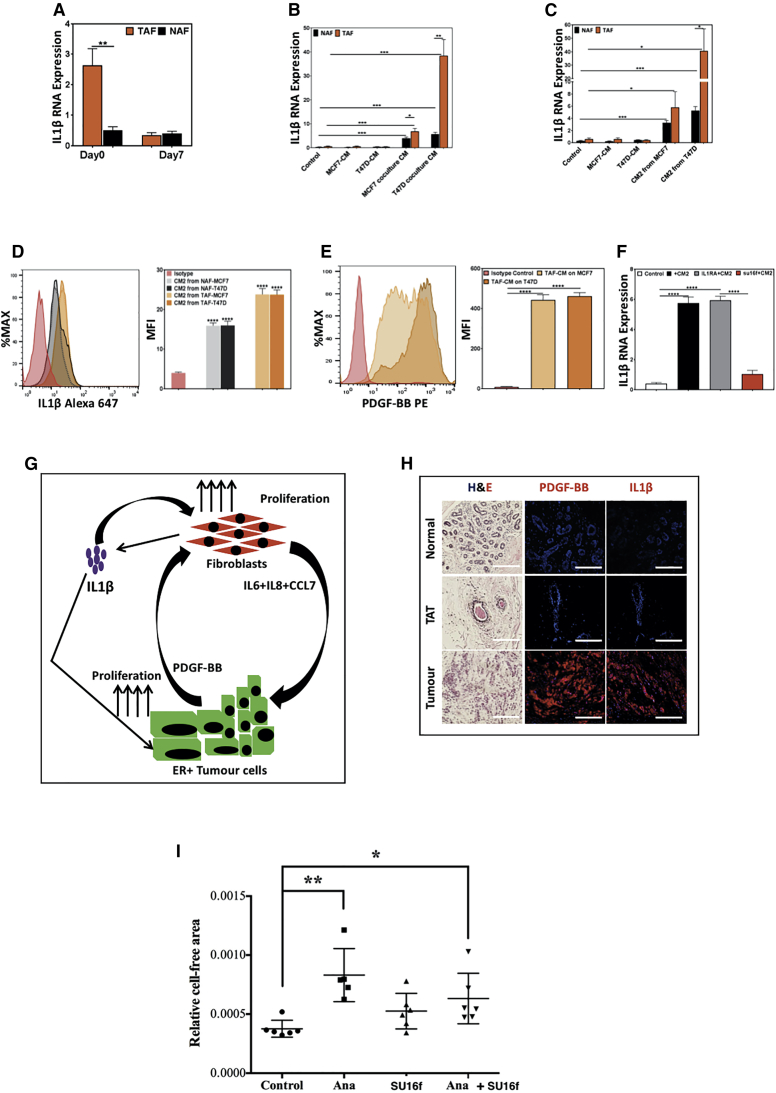
Crosstalk between TAFs and ER^+^BCCs Results in IL1β Production by TAFs (A) *IL1β* transcript expression was measured in TAFs and NAFs immediately after isolation (day 0) and after 7 days of 2-dimensional (2D) adherent cultures. (B) NAFs and TAFs were treated with conditioned media (CM) from MCF7- and T47D-only cultures or from the 2D co-culture with NAFs or TAFs for 6 h, and *IL1β* transcript levels in the fibroblasts were quantified via qPCR. (C) NAFs and TAFs were treated with CMs from MCF7 and T47D or (CM2, from Figure 4C) for 6 h, and *IL1β* transcript levels were measured. (D) IL1β protein expression was measured in NAFs and TAFs following treatment with CM2 for 48 h by intracellular flow cytometry. (E) PDGF-BB protein expression was measured in MCF7 and T47D following treatment with CM from TAFs for 48 h by intracellular flow cytometry. (F) *IL1β* transcript expression was measured in TAFs following treatment with CM2 for 6 h with or without 48-h pre-incubation with IL1RA or SU16f. All data are represented as bar graphs with mean ± SEM from three independent experiments. (G) Graphical summary of the crosstalk between fibroblasts and breast cancer cells resulting in IL1β secretion from the fibroblasts. (H) Representative H&E-stained sections and immunofluorescent images showing PDGF-BB and IL1β protein expression from three individual ER^+^ breast cancer tumors, matched tumor-adjacent, and three healthy normal breast tissues. (I) Tumor progression was monitored in an orthotopic mouse breast cancer model using TAFs and MCF7 cells. Mice were either treated with Anakinra (Ana), SU16f (Su), Ana + Su, or vehicle control. Cell-free areas of H&E sections from each tumor was obtained (Figure S5I), normalized to the total area for each xenograft section, and plotted in dot plots. Scale bars, 200 μm. ∗p < .05, ∗∗p < .005, ∗∗∗p < .0005, and ∗∗∗∗p < .00005.

To further explore the paracrine mechanisms involved in PDGF-BB production from BCCs, we compared the NAF-CM with the MCF7- and T47D-CM and identified four cytokines (CCL7, IL6, IL8, and GROα) that were uniquely secreted by NAFs (Figure 5A and Table S2). Interestingly, IL6 and IL8 together, but not alone, as well as CCL7 stimulated (2.6 ± 0.6-fold and 6.6 ± 1.5-fold, respectively) *PDGFB* gene expression in the primary ER^+^BCCs; however, CCL7 is the major inducer of PDGF-BB production by ER^+^BCCs (Figures 5B and 5C). GROα had no effect on PDGF-BB production. We found that IL6, IL8, and CCL7 are expressed at significant levels in the normal breast, ER^+^ breast tumors, and the matching tumor-adjacent breast tissue (Figure 5D). These data suggest that the secretion of these cytokines does not require the presence of ER^+^BCCs. However, the tumor microenvironment consists of activated TAFs that do not show sustained IL1β secretion (Figure 6A), suggesting that TAFs require the presence of ER^+^BCCs to maintain IL1β secretion (Figure 6B). Interestingly however, TAFs induced MCF7 and T47D proliferation more significantly than NAFs in co-cultures (Figures S5A and S5B). To this end we found that similar to NAFs TAF-CM induces PDGF-BB secretion in MCF7 and T47D cells, which in turn causes IL1β production from TAFs (Figures 6C–6F and S5A–S5F).

### TAFs and Infiltrating Immune Cells Create an IL1β-Enriched Microenvironment in ER^+^ Breast Tumors

Our data describe a paracrine signaling mechanism between ER^+^BCCs, NAFs, and TAFs that result in the secretion of IL1β from fibroblasts without requirement for immune cell response, leading to tumor cell proliferation (Figure 6G). As predicted by our model, PDGF-BB and IL1β can only be detected in the tumor tissue, indicating that the cross talk between the TAFs and the ER^+^BCCs and not the normal breast epithelial cells results in PDGF-BB and IL1β production (Figure 6H). Next, we examined if *PDGFB* and *IL1β* expression are predictive of primary ER^+^ breast cancer prognosis using SurvExpress online database. Interestingly, *IL1β* and *PDGFB* expression significantly separated the high-risk group from the low-risk group (concordance index = 54.9, log rank equal curves p = 0.0052, risk group hazard ratio = 1.6 [confidence interval 1.15–2.27]) and that both *IL1β* and *PDGFB* were more significantly expressed in the high-risk group compared with the low-risk group (Figures S5G and S5H). These observations suggest that the paracrine signaling described here could take place in the tumor environment and has clinical relevance to tumor progression. To further explore this possibility, we created the tumor microenvironment in NSG (NOD-scid IL2Rgamma^null^) mice using TAFs and the ER^+^ MCF7 BCCs and examined the impact of blocking IL1β (Anakinra) and PDGF ( SU16f) signaling on tumor growth *in vivo*. We observed that xenografts generated in mice treated with Anakinra alone or with combination of Anakinra and SU16f showed statistically significant but modestly decreased tumor cell density compared with the xenografts obtained from untreated control mice (Figures 6I and S5I). SU16f-treated xenografts did not show any significant increase in necrotic or cell-free areas when compared with the untreated control xenografts. Notably, the intra-nipple injection model used allowed for confined growth within the mammary ducts. Accordingly, no difference in tumor weight was observed among the different treatment groups; hence alterations in tumor histology were not due to dramatic differences in tumor volume (data not shown).

**Figure 7:**
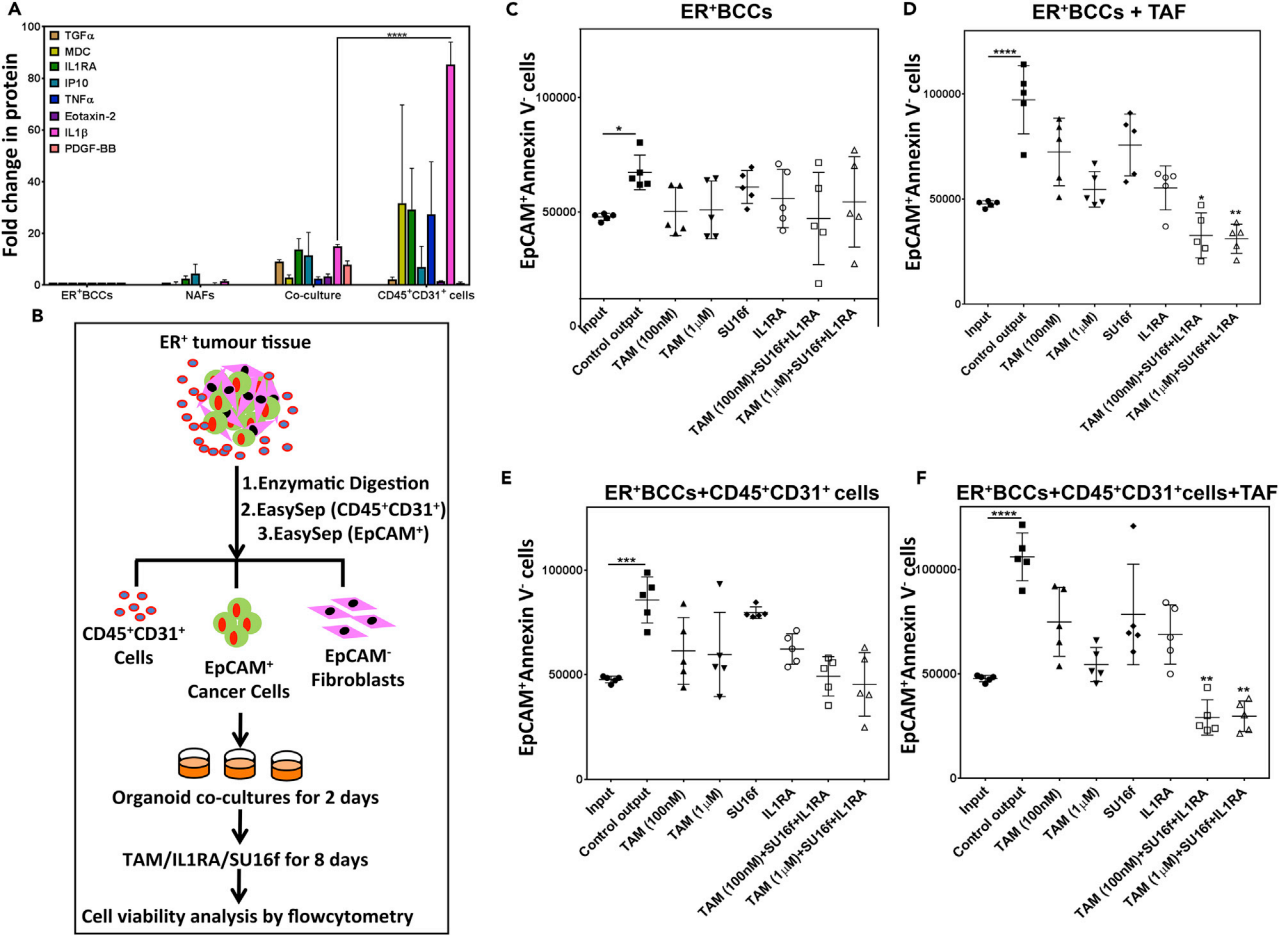
Deconstruction and Reconstruction of Tumor Microenvironment (A) Cytokine ELISA array was used to examine cytokines present in the CM obtained from organoid cultures of primary ER^+^BCCs, matched tumor-associated CD45^+^CD31^+^ cells, NAFs, and NAFs-ER^+^BCCs co-cultures. Cytokines that were secreted at high concentration by the NAF-ER^+^BCCs co-cultures, as well by the CD45^+^CD31^+^ cells, are shown (Table S3). Average from three biological replicates and SEM are plotted as bar graphs where average cytokine levels in breast cancer cells are set to 1. (B–F) (B) Experimental outline for (C–F). The ER^+^BCCs (C), ER^+^BCCs with BCC-associated fibroblasts (TAFs) (D), ER^+^BCCs with matching BCC-associated CD45^+^CD31^+^ cells (E), and all three cell types together (F) were put into organoid cultures for 2 days and then treated with IL1RA, SU16F, and tamoxifen (Tam) as indicated for additional 8 days, and the number of viable (AnnexinV^−^) EpCAM^+^BCCs was determined by flow cytometry. The data represented as dot plots from primary ER^+^ breast cells obtained from five individual tumors, and average cell numbers and SEM are plotted (∗p < .05, ∗∗p < .005, ∗∗∗p < .0005, and ∗∗∗∗p < .00005, statistical significance was tested against the input controls).

In the tumor microenvironment, however, in addition to TAFs (Figure S6A), the tumor-infiltrating immune cells and the vascular endothelial cells also contribute to tumor cell proliferation. To examine the contribution of the immune and blood vessel cells to tumor cell proliferation, we utilized the cytokine ELISA array platform to analyze the secreted cytokine profile of CD45^+^CD31^+^ cells obtained from three primary ER^+^ breast tumor samples after 10 days in 3D organoid culture (Figures 7A, S6B, and S6C). The analysis identified that some of the same cytokines secreted by the NAF and ER^+^BCC co-cultures were also secreted at significantly higher levels (Table S3) by the tumor-associated CD45 ^+^31^+^ cells. Compared with the ER^+^BCC-NAF organoid co-cultures, the tumor-associated CD45^+^CD31^+^ cells secrete 5.7 ± 0.48-fold more IL1β, 1.5 ± 2-fold CCL7, 1.2 ± 0.2-fold IL6, and 1.1 ± 0.02-fold IL8 (Figure 7A and Table S3). The immunophenotyping analysis of the tumor-associated CD45 ^+^ 31^+^ cells revealed a higher presence of T cells and lower but detectable levels of monocytes, B cells, and natural killer cells (Figure S6D), which matched the secreted cytokine profile of these cells (Figure S6E).

### Disrupting the ER^+^BCC-TAF Paracrine Signaling Significantly Amplifies Tamoxifen Efficacy

Our data indicate that the paracrine signaling between the ER^+^BCC and fibroblasts cooperates with the CD45^+^CD31^+^ cells in the tumor microenvironment to create an IL1β-enriched niche that augments tumor cell proliferation. We therefore examined if the disruption of IL1β production and signaling in the tumor microenvironment would augment the cytostatic effect of Tam on the ER^+^BCCs. For this purpose, we reconstructed the tumor microenvironment in organoid cultures using the primary ER^+^BCCs, TAFs, and CD45^+^CD31^+^ cells separately obtained from the same original tumor (Figure 7B). Tumor-associated CD45^+^CD31^+^ cells showed a small (20%–30%) decrease in cell number in these organoid cultures over 10 days (Figure S6B). We examined the effective dose of Tam through its ability to decrease MCF7 cell proliferation in organoid cultures (Figure S6F) and found that 100 nM Tam was ineffective in reducing MCF7 cell proliferation, whereas 1 μM Tam significantly reduced proliferation of these, and we therefore chose these concentrations for our experiments. To examine the contribution of the TAFs and CD45^+^CD31^+^ cells to tumor cell proliferation, we quantified AnnexinV^−^EpCAM^+^ cell numbers in organoid cultures initiated with primary ER^+^BCCs alone or in co-cultures with TAFs, CD45^+^CD31^+^, or all three cell types together. ER^+^BCCs expanded 1.3 ± 0.1-fold in the organoid cultures. However, ER^+^BCCs showed further proliferation in co-cultures with TAFs (1.96 ± 0.3) and CD45^+^CD31^+^ (1.72 ± 0.2), but no additional proliferation was detected in co-cultures consisting of all three cell types (Figures 7C–7F). In contrast to MCF7 cells (Figure S6E), 100 nM Tam prevented the expansion of ER^+^BCCs in all the cultures. A selective PDGF-BB receptor inhibitor SU16f and an IL1R1 antagonist IL1RA, showed similar effects as Tam in all the co-cultures, although the results were variable. However, the ER^+^BCC proliferation in co-cultures were significantly curtailed when organoids were treated with Tam, SU16f, and IL1RA, suggesting that these blockers work synergistically with Tam. In TAF-ER^+^BCC organoid co-cultures, SU16f and IL1RA were as effective as Tam in decreasing ER^+^BCC proliferation.

## Discussion

In this report, we describe a fibroblast-initiated paracrine signaling mechanism that enhances ER^+^BCC proliferation. We show that cytokines constitutively secreted by NAFs and TAFs alike (CCL7 and/or IL6 and IL8) result in increased expression and secretion of PDGF-BB from primary ER^+^BCCs but not the normal breast epithelial cells. In this regard, CCL7 makes the most significant contribution toward the release of PDGF-BB from ER^+^BCCs. CCL7 is a ligand for CCR1–3 receptors, among which expression of CCR3 has recently been shown to be associated with luminal-type breast cancers (Gong et al., 2016). Also, the interaction between CCL7 and CCR3 has been shown to promote colon cancer cell metastasis via ERK-JNK signaling (Lee et al., 2016). The role of IL6 and IL8 in breast cancer progression has been well studied in that both cytokines increase the invasiveness and the metastatic potential of ER^+^ and ER^−^ breast cancers by activating signaling through their receptors, gp130 and CXCR1 (Ginestier et al., 2010, Iliopoulos et al., 2010, Korkaya et al., 2011, Sasser et al., 2007, Sheridan et al., 2006, Studebaker et al., 2008). Previous reports indicate that PDGF-BB increases the expression of IL6 and IL8 in different cell types (van Steensel et al., 2010). Here we show that increased PDGF-BB levels increases in ER^+^BCCs due to CCL7, IL6, or IL8 cytokine signaling. IL6, IL8, and CCL7 presumably increase PDGF-BB expression by activating their cognate receptors, which have been previously shown to be expressed in ER^+^BCCs. The PDGF-BB secreted from ER^+^BCC signals through its receptor PDGFRβ to induce IL1β production in the normal and activated fibroblasts without the requirement for IL1β receptor IL1R1 activation, suggesting that fibroblast secretion of IL1β is not regulated through a feedforward mechanism. Signaling through the PDGFRβ activates the STAT3 and ERK signaling, which have previously been reported (O'Brien et al., 1999, Tian et al., 2017) to induce IL1β transcripts and protein levels. These signaling pathways therefore are the most likely mechanism of PDGF-BB-induced IL1β production from NAFs and TAFs. We also show that although IL1β suppresses the proliferation of normal breast epithelial progenitors, it creates a pro-oncogenic environment by activating proliferation signals in ER^+^BCCs. Therefore, our data suggest that features distinguishing the ER^+^BCCs from the normal breast epithelial cells are the ability to take advantage of fibroblast-secreted factors to produce PDGF-BB and to utilize IL1β signaling as a pro-proliferative signal. Our orthotopic mouse tumor data suggest that blocking IL1β could potentially decrease tumor volume in xenografts initiated with TAFs and BCCs; however, additional data are required to provide stronger support for this observation.

Our findings suggest that in patients with breast cancer undergoing breast-conserving surgeries, any residual BCCs could take advantage of cytokines constitutively secreted by the fibroblasts and initiate the paracrine signaling cascade described here to transform the normal breast tissue niche into an IL1β-enriched niche that supports their proliferation. Previously it was demonstrated that NAFs are able to support cancer cell proliferation *in vivo*; however, they do so at much lower efficiency than TAFs (Chatterjee et al., 2018, Olumi et al., 1999). It is therefore inviting to hypothesize that in normal breast tissue, epigenetic or accumulating mutational events could enable the normal epithelial cells to utilize the CCL7 or IL6 and IL8 signaling pathways to produce PDGF-BB, ultimately inducing IL1β release from fibroblasts. However, the ability to produce PDGF-BB in the presence of these cytokines, on its own, is not sufficient to promote the proliferation of breast epithelial cells because we show that IL1β significantly diminishes the proliferation of normal breast epithelial progenitors.

Using cytokine array platform, we found that in the tumor microenvironment the immune cells, in particular monocytes/macrophages, are the most significant sources of IL1β when compared with fibroblasts. We therefore have demonstrated the clinical significance of our findings by assessing the individual contributions of TAFs and immune cells in tumor response to endocrine therapy. By reconstructing the tumor microenvironment using ER^+^BCCs, TAFs, and the immune cells from the same primary tumors, we demonstrate that the effectiveness of endocrine therapy (Tam) can be significantly augmented in the presence of IL1R1 and PDGFRβ blockers. This observation is particularly interesting because examining the contribution of tumor-infiltrating leukocytes (TILs) to ER^+^BCC proliferation and response to endocrine therapy cannot be examined through the other available models to study ER^+^ tumor microenvironment. Although primary tumors can be expanded through several passages in immunodeficient mice (i.e., PDX), only 4%–6% of primary ER^+^ tumors form xenografts. Moreover, it is unlikely that activated TILs would be propagated in passaged xenografts. On the other hand, the ER^+^BCC lines (MCF7 and T47D) can readily form xenografts in immunodeficient mice. However, these cell lines have been generated from metastatic breast cancer tumors, which might not be an accurate representation of primary ER^+^ breast cancer tumors. Also, owing to major histocompatibility complex molecule incompatibility, TILs cannot be included in these xenografts. Finally, our results suggest that patients diagnosed with malignant ER^+^ breast cancer may benefit from combination therapy consisting of IL1R1 and PDGFRβ blockers to enhance the effectiveness of endocrine therapies such as Tam.

### Limitations of the Study

In our study we provide evidence that regularly secreted cytokines from fibroblasts result in creation of an IL1β-enriched environment that supports ER^+^BCC proliferation. Such paracrine signaling might be important to tumor progression and therapy response. However, there are limitations to our study due to some experimental constraints. The impact of blocking IL1β and PDGF-BB signaling on tumor growth *in vivo* was rather modest. However, the *in vivo* model we used did not ensure that fibroblasts and tumor cells will be confined to the xenografted site in the mammary gland. This issue could be addressed by using PDXs where tumor cells and fibroblasts are present in the primary breast tumor section. Moreover, the use of combination therapy that includes endocrine therapy along with IL1β and PDGF-BB signaling blockers might provide more definitive data with respect to decreased tumor cell density.

Second, in our patient-derived organoid cultures, we used CD45+ immune cells and the CD31+ vascular endothelial cells, which did not allow us to evaluate the contribution of each cell type to tumor cell growth and response to therapy separately. Owing to the low frequency at which these cells are present in primary breast tumors, obtaining each of these cell types separately in sufficient numbers proved difficult.

## Methods

All methods can be found in the accompanying Transparent Methods supplemental file.

## Supplemental Information

Document S1. Transparent Methods and Figures S1–S6

Table S1. Cytokine Array Data for Conditioned Media Collected from ER^+^ BCCs and NAFs Grown Either Alone or in Co-cultures, Related to Figure 1 Primary estrogen receptor-positive (ER^+^) breast cancer cells (BCCs) were placed in Matrigel cultures either alone or in co-cultures with normal-associated fibroblasts (NAFs). ELISA array was used to quantify secreted cytokine levels in the conditioned media collected from these cultures. The numbers represent observed cytokine concentration (ObsConc) in pg/mL. Data are obtained from three independent ER^+^ tumors and NAF samples. The numbers in the parenthesis refer to the position of the antibodies to detect a specific cytokine in the ELISA array grid.

Table S2. Comparative Cytokine Array Analysis of CM1 and CM2, Related to Figures 4 and 5 ELISA array platform was used to quantify and compare the secreted cytokine levels in conditioned media 1 (CM1) and CM2 obtained as described in Figure 4C. Average secreted cytokine levels and standard deviations from three independent MCF7 and T47D cells are shown in bar graphs.

Table S3. Secreted Cytokines by Tumour-Associated CD45^+^CD31^+^ Cells, Related to Figure 7 From three different estrogen receptor-positive (ER+) breast cancer tumor samples, the immune cells (CD45^+^) and the endothelial cells (CD31^+^) cells were removed and placed in Matrigel cultures. The secreted cytokine levels in these cultures were quantified using an ELIA array platform. The numbers in the table are in pg/mL.
