# Supplementary material for: Paracrine Crosstalk between Fibroblasts and ER+ Breast Cancer Cells Creates an IL1β-Enriched Niche that Promotes Tumor Growth
Source: iScience. 2019 Jul 24;19:388–401. doi: 10.1016/j.isci.2019.07.034 (PMC6706609; doi:10.1016/j.isci.2019.07.034)

ISCI, Volume *19*

Supplemental Information

Paracrine Crosstalk between Fibroblasts and ER$^+$ Breast Cancer Cells Creates an IL1β-Enriched Niche that Promotes Tumor Growth

Sumanta Chatterjee, Vasudeva Bhat, Alexei Berdnikov, Jiahui Liu, Guihua Zhang, Edward Buchel, Janice Safneck, Aaron J. Marshall, Leigh C. Murphy, Lynne-Marie Postovit, and Afshin Raouf

Transparent Methods

Tissue collection and processing

Estrogen receptor positive (ER^+) breast tumours (>2cm) and matching tumour-adjacent tissue (TAT) samples were obtained from patients undergoing mastectomy surgery without any prior treatment. TAT samples were obtained 3-6 cm away from the primary tumour margin and declared disease-free and histologically benign by a pathologist. Contralateral tumour-free breast tissue was also obtained from patients with ER^+ tumours undergoing prophylactic bilateral mastectomy. As a source of normal breast tissue, reduction mammoplasty samples were obtained. Samples were transported to the laboratory in transport medium as previously described (Basak et al., 2015; Chatterjee et al., 2018; Chatterjee et al., 2015) for further processing. All samples were obtained with written informed patient consent according to protocols approved by the University of Manitoba's Research Ethics Board. Tissue samples were dissociated enzymatically for 16 hours in dissociation media as described previously. Cell pellets were re-suspended in 6% dimethylsulfoxide (DMSO)-containing fetal bovine serum (FBS)-supplemented medium for liquid nitrogen storage.

Isolation of different subset of cells from the breast tumours

Single cell suspensions from fresh or frozen tumour samples were depleted of immune and endothelial cells by magnetically separating $CD45^+CD31^+$ (Lin-) cells using EasySep™ Human Biotin Selection Kit (StemCell Tech.) and were cryogenically preserved in liquid Nitrogen. Subsequently, the $EpCAM^+$ breast cancer cells were isolated using EasySep™ Human EpCAM Positive Selection Kit (StemCell Tech.). Isolated $EpCAM^+$ breast cancer cells (BCCs) were either cryogenically preserved or used right away. Before freezing, viability for each cell fraction was assessed by propidium iodide (PI) staining and flow cytometric analysis. The $EpCAM^-$ cell fraction was used to generate tumour-associated fibroblasts (TAFs) lines as described previously (Chatterjee et al., 2018).

Patient-derived organoid cultures

50 µl of liquid growth factor-reduced matrigel (BD Biosciences) was placed in each well of a 96-well plate and 50 µl of PBS added on top to prevent evaporation while the gels were allowed to polymerize at 37°C for 30 minutes in a humidified chamber. Between $3x10^4$-$5x10^4$ cells from the primary $EpCAM^+$ BCCs and *in vitro* expanded fibroblasts were mixed with 200µl organoid media (SF7 growth media (Stingl et al., 2001)supplemented with 10µM Y27632 and 10µM SB431542) (both from StemCell Tech.) and placed on top of polymerized matrigels either alone or together at 1:1 ratio with NAFs or TAFs and plates were transferred to a humidified 37°C, 5% CO_2 incubators

at ambient O_2. For the purposes of these experiments, we successfully generated 11 patient-derived organoids from 17 individual ER+ breast tumour samples.

The medium was changed every 3 days and after 10 days, gels were dissolved with dispase and organoids were made into single-cell suspension as described previously (Chatterjee et al., 2018). For some experiments, organoid cultures also contained $CD45^+CD31^+$ cell fraction from the tumours at 1:1:1 ratio (50,000 of each cell type) with $EpCAM^+$ BCCs and TAFs obtained from the same original tumour. In some experiments, organoid media were supplemented with recombinant CCL7 (1-10ng/mL), IL6 (1-10ng/mL), IL8 (1-10ng/mL), TGFα (1-10ng/mL), MDC (1-10ng/mL), GROα (1-10ng/mL), IL1RA (1-10ng/mL), IL1β (10pg-1ng/mL), and PDGF-BB (500pg-10ng/mL) (all from Sigma). Some co-cultures were treated with 4-hydroxytamoxifen (Tam, at, 100nM and 1µM, Tocris Biosciences), IL1RA (IL1R1 receptor antagonist, 100ng/mL, Biolegend) and SU16f (selective PDGFRβ blocker, 10µM, Tocris Biosciences) (Cheng et al., 2011; Jiang et al., 2017; Lee et al., 2014).

In some experiments MCF7 cells were placed in organoid cultures with SF7 growth media and After 48 hr, media were replaced with fresh media containing different concentrations (50nM-1µM) of Tam and cultured for an additional 8 days with media changes every 3 days. Subsequently, gels were dissolved and made into single-cells as described (Basak et al., 2015; Chatterjee et al., 2018) and cell viability determined via PI dye retention using flow cytometry.

Primary fibroblast cultures

Fibroblasts from the Tumour (TAFs), TAT (TAT-Fs), and normal breast reduction (NAFs) samples were obtained by expanding the $EpCAM^-$ subset of dissociated tumour cells in adherent 2 dimensional (2D) cultures as described before (Chatterjee et al., 2018). Briefly, $EpCAM^-$ cells were cultured in DMEM/F12 media supplemented with 10% FBS at 37°C and cultured to 70-75% confluency. All fibroblast lines were passaged at least twice to obtain near homogeneity and presence of mesenchymal markers (CD73, CD90, CD105, CD13 and FSP1/S100A4) (all from BD-Biosciences) and absence of epithelial (EpCAM) (StemCell Technologies), endothelial (CD31) (e-biosciences) and immune (CD45) (BD-Biosciences) cell markers was examined by flowcytometry.

Breast cancer cell lines

ER^+ breast cancer cell lines MCF7 and T47D and the triple negative MDA-MB-231 cells were maintained in 5% FBS containing DMEM or 10% FBS containing RPMI or 10% FBS containing DMEM media growth media respectively. MCF7 and T47D cell lines were authenticated recently (October, 2016) using STR analyses (Genetica Cell Line Testing, Labcorp, Burlington, NC, USA). All experiments were carried out using stocks between passages 2-5. All cells were grown upto 70-75% confluency before passaging. For some experiments, MCF7 and T47D cells were co-cultured with NAFs, TAT-Fs, TAFs, or contralateral non-tumour containing breast-fibroblasts (CNTB-Fs) at 1:1 ratio ($5x10^4$-$1x10^5$ of each cell type) for up to 10 days in 2D adherent cultures.

For other experiments, MCF7 and T47D cells were grown in 3D matrigels and treated with recombinant TGFα, MDC, IL1RA, IL1β and PDGF-B (all from sigma) for 8 days.

Conditioned media (CM) collection

Primary EpCAM$^+$ BCCs (1x10^5) were placed in organoid cultures either alone or with NAFs or TAFs (1x10^5) and after 48 hr medium was replaced with 100 µl of fresh medium which was then collected either after 48 hr or 8 days (as control for long-term organoid cultures). The collected media (conditioned media, CM) were centrifuged for 5 mins at 1200 rpm and supernatants were stored at -80°c. Fibroblast-only conditioned media were also collected. For some experiments CM were obtained from the CD45$^+$CD31$^+$ cell fraction of breast tumours grown in matrigel for 8 days. For some other experiments, MCF7 and T47D cells were cultured in 2D either alone or with different fibroblasts for upto 75% confluence when fresh medium was added and CM were collected after 48hr.

Cytokine ELISA array

CM collected after 8 days from the EpCAM$^+$ BCCs and NAFs in organoid cultures either alone or in co-culture were sent to Eve Technologies for 65-plex human cytokine/chemokine enzyme-linked immunosorbent assay (ELISA) analysis. For some experiments, cytokine array analysis was done on CM collected from the CD45$^+$CD31$^+$ cell fraction of the tumours grown in matrigel for 8 days. For experiments described in Fig. 4C, MCF7, T47D, and NAF cells were grown in 2D adherent cultures and CM were collected from each culture after 48hr (Fig. 4C, CM1). TAF-CM was placed on MCF7 or T47D for 48hr (Fig. 4C, CM2). CM1 and CM2 samples were sent for cytokine/chemokine ELISA array analysis (Table S2). Each cytokine/chemokine examined was accompanied with a standard curve and cytokine expression values are the average of 3 biological replicates.

Colony-forming cell assays

Colony-forming cell (CFC) assays were performed as previously described (Basak et al., 2015; Chatterjee et al., 2018; Chatterjee et al., 2015). Briefly, 5,000 Lin- cells from breast reduction samples or dissociated organoids from matrigels were plated together with 80,000 irradiated mouse NIH 3T3 cells in SF7 medium supplemented with 5% FBS. After 7–10 days, colonies were fixed with a 1:1 (vol/vol) mixture of methanol and acetone on ice and then stained with crystal violet (Sigma). Colony types and numbers were obtained using a bright field microscope.

Flow cytometric analyses

Single-cell suspensions obtained from the $EpCAM^+$, $CD45^+CD31^+$ subset of ER^+ primary tumours were pre-blocked with 2% FBS-containing Hank's Balanced Salt Solutions (HBSS) supplemented with 10% human serum for 15 minutes. The $EpCAM^+$ cells were labelled with anti-human EpCAM (StemCell Technologies) and MUC1 (Millipore) antibodies and the $CD45^+CD31^+$ cells were stained with LIVE/DEAD™ Fixable Aqua Dead Cell Stain Kit (Thermo Fischer) and anti-human CD45, CD3, CD19, CD14 and CD56 antibodies (all from BD Biosciences). NAFs and TAFs were stained with anti-human CD73, CD90, CD105, CD13 (all from BD Biosciences) and IL1R1 (Invitrogen) antibodies. For some experiments, single cell suspensions from organoid-enriched fractions were labeled with anti-human CD49f (Biolegend) and EpCAM antibodies. To obtain MCF7 and T47D cell numbers in fibroblast co-cultures, cells were stained with anti EpCAM antibody. For all experiments, propidium iodide (PI, Sigma) exclusion was used to identify viable cells.
Intracellular flow cytometry was performed as described (Basak et al., 2015) to detect CK5, CK14, CK8/18, αSMA, p63 and ERα expression in the $EpCAM^+$ BCCs and the expression of FSP1 in the fibroblasts. For analyzing cytokine secretion (IL1β, PDGF-BB, CCL7, IL6 and IL8), cells were first incubated with Brefeldin A (e-Biosciences) for 6 hr before fixing and stained with different primary antibodies. The flow cytometry data were analyzed using the FlowJo software (BD-Biosciences).

Quantitative real-time PCR

Total RNA was extracted from fresh, FACS-sorted non-cultured or cultured cells using the Trizol reagent (Invitrogen). cDNA was prepared from 1 μg of RNA using the Maxima cDNA synthesis kit (Thermo Fisher) and then used as a template for PCR. Transcript expression of specific genes was obtained using gene-specific primers. Relative expression levels of specific transcripts were calculated by normalizing to housekeeping genes (*GAPDH, TFRC* and *HPRT*) transcript levels.

Western blot analysis

Fibroblasts were grown in 2D culture to 70-75% confluency and protein extracts were prepared using 2% sodium dodecyl sulfate (SDS) buffer with complete protease inhibitor tablets (Roche Diagnostics). Western Blots were carried out using standard protocols with 70-90 μg of total protein. Protein expression was determined using anti PDGFRα (Santa Cruz Biotech.), PDGFRβ (Santa Cruz Biotech.), STAT3 (Cell Signaling Tech.), phosphorylated STAT3 (pSTAT3, Cell Signaling Tech.), and beta actin (Sigma) antibodies by chemiluminescence. The expression level of each protein was determined using beta actin expression as loading control.

Clinical outcome and gene expression analysis

To assess the relationship between *IL1β* and *PDGFB* gene expression and patients' risk/survival in The Cancer Genome Atlas (TCGA) invasive breast carcinoma cohorts (962 cases; build July 2016), we used the SurvExpress tool (http://bioinformatica.mty.itesm.mx:8080/Biomatec/SurvivaX.jsp). A prognostic index (PI) was generated from the most up-to-date TCGA invasive breast carcinoma data set using *IL1β* and *PDGFB* gene expression. PI is the linear component of the Cox model, PI=β1x1+ β2x2+...+βpxp, where xi is the gene expression value and the βI can be obtained from the Cox fitting. Each βI can be interpreted as a risk coefficient. The risk groups are generated by dividing the ranked order PI values into 2 groups, low risk and high risk group (i.e. higher PI values). This achieved by using the medial PI score to divide the data set into 2 groups. The log-ranked test of differences between risk groups, hazard ratio estimate, and gene expression in high and low risk groups was obtained. For *IL1β* and *PDGFB*, the software determined the prognostic index values which were used to divide the dataset into high and low-risk groups; overall Survival was used as clinical endpoint.

Animal experiments

ER+ MCF7 breast cancer cells ($1x10^6$ cells) and tumour-associated fibroblasts ($1x10^6$ cells) were mixed and injected intraductally in to 6-8 weeks old non-obese diabetic (NOD)-scid IL2Rgamma^{null} (NSG) female mice with estrogen pellet implants (17β-ESTRADIOL pellets (1.7 mg, 60-day release, Innovative Research of America as in (Quail et al., 2012)). All experiments involving animals were approved by the Animal Use Subcommittee at the University of Alberta (AUP00001288 and AUP00001685). Two days after injection, mice (6 mice per experimental arm) were treated daily with an IL1β receptor blocker Anakinra (400mg/kg of body weight/per day injected subcutaneously) or a PDGF receptor blocker SU16F (10mg/ kg of body weight/day via gastric gavage) or combination of both drugs or DMSO as vehicle control. After 2 weeks, mice were sacrificed, tumour xenografts were excised and weighed, and formalin-fixed sections from each tumour was stained with hematoxylin and eosin (H&E).

Cell density quantification of H&E sections

Quantification of cell density (i.e. cell-free areas) in the H&E sections from the tumour xenografts was done using QuPath v0.1.2 (https://qupath.github.io/QuPath-v0.2.0) software in conjunction with the ImageJ (https://imagej.net) essentially as described (Bankhead et al., 2017). The software's manual annotation tool was used to select the total tumour area and the *SLIC superpixel segmentation* command was applied to subdivide the annotated region into 'superpixels' (yellow lines, Supplementary Figure 5I). Intensity features were obtained for each superpixels along with Haralick texture features(Bankhead et al., 2017). A two-way random trees classifier was trained to distinguish between area with cells (superpixel segments) and cell-free area (no cell nucleus). The regions with cells and cell-free area were manually curated for each H&E image and annotated. This classification was then applied to all H&E images. The ratio of cell-free area to total area was calculated and used as a surrogate measure of cell density within each xenograft.

Statistical Analysis

The ANOVA and student T-tests were performed using the GraphPad Prism 7.02 program (San Diego, CA).

Supplementary References

Bankhead, P., Loughrey, M.B., Fernandez, J.A., Dombrowski, Y., McArt, D.G., Dunne, P.D., McQuaid, S., Gray, R.T., Murray, L.J., Coleman, H.G.*, et al.* (2017). QuPath: Open source software for digital pathology image analysis. Scientific reports *7*, 16878.

Basak, P., Chatterjee, S., Weger, S., Bruce, M.C., Murphy, L.C., and Raouf, A. (2015). Estrogen regulates luminal progenitor cell differentiation through H19 gene expression. Endocr Relat Cancer *22*, 505-517.

Chatterjee, S., Basak, P., Buchel, E., Safneck, J., Murphy, L.C., Mowat, M., Kung, S.K., Eirew, P., Eaves, C.J., and Raouf, A. (2018). Breast Cancers Activate Stromal Fibroblast-Induced Suppression of Progenitors in Adjacent Normal Tissue. Stem cell reports *10*, 196-211.

Chatterjee, S., Laliberte, M., Blelloch, S., Ratanshi, I., Safneck, J., Buchel, E., and Raouf, A. (2015). Adipose-Derived Stromal Vascular Fraction Differentially Expands Breast Progenitors in Tissue Adjacent to Tumors Compared to Healthy Breast Tissue. Plastic and reconstructive surgery *136*, 414e-425e.

Cheng, F., Pekkonen, P., Laurinavicius, S., Sugiyama, N., Henderson, S., Gunther, T., Rantanen, V., Kaivanto, E., Aavikko, M., Sarek, G.*, et al.* (2011). KSHV-initiated notch activation leads to membrane-type-1 matrix metalloproteinase-dependent lymphatic endothelial-to-mesenchymal transition. Cell host & microbe *10*, 577-590.

Jiang, Y., Berry, D.C., Jo, A., Tang, W., Arpke, R.W., Kyba, M., and Graff, J.M. (2017). A PPARgamma transcriptional cascade directs adipose progenitor cell-niche interaction and niche expansion. Nature communications *8*, 15926.

Lee, E., Pandey, N.B., and Popel, A.S. (2014). Lymphatic endothelial cells support tumor growth in breast cancer. Scientific reports *4*, 5853.

Quail, D.F., Zhang, G., Walsh, L.A., Siegers, G.M., Dieters-Castator, D.Z., Findlay, S.D., Broughton, H., Putman, D.M., Hess, D.A., and Postovit, L.M. (2012). Embryonic morphogen nodal promotes breast cancer growth and progression. PLoS One *7*, e48237.
Stingl, J., Eaves, C.J., Zandieh, I., and Emerman, J.T. (2001). Characterization of bipotent mammary epithelial progenitor cells in normal adult human breast tissue. Breast Cancer Res Treat *67*, 93-109.

Supplementary Figure Legends

Supplementary Figure 1 (A-G) related to Figure 1. Maintenance of primary ER^+ breast cancer cells in organoid cultures and proliferation of MCF7 and T47D in co-cultures

(A) Expression of basal and epithelial cell markers was measured in the primary $EpCAM^+$ breast cancer cells (BCCs) before and after organoid culture for 10 days. Data from 3 different samples are shown as dot plots. (B) Expression of mesenchymal and fibroblast markers was measured in fibroblasts obtained from normal breast reduction tissues after 2 passages. Data from 5 different samples are shown as dot plots. (C) $EpCAM^+$ BCCs obtained from 11 different ER^+ tumour samples were grown in organoid cultures for 10 days and cell numbers are plotted as box and scatter plots. (D) Normal-associated fibroblast (NAF) cell numbers before and after 10-day organoid cultures are shown as box and scatter plot. Data from 6 samples were plotted. (E) 2D adherent co-cultures were initiated with estrogen receptor (ER^+) breast cancer cell lines (MCF7, T47D) and NAFs or contralateral non-tumour breast tissue (CNTB-F) and number of viable $EpCAM^+$ cancer cells (E & F) or the $EpCAM^-$ fibroblasts (G) were quantified by flow cytometry at the beginning (input) and after 3, 5 and 10 days (output). Average cell numbers and standard error of the mean (SEM) from 3 independent experiments are represented in the line graphs. (***P < .0005 and ****P < .00005)

Supplementary Figure 2 (A-F) related to Figure 2. IL1β inhibits normal breast epithelial progenitor expansion. Transcript expressions of *IL1β* target genes were assessed by qRT-PCR in (A) normal-associated fibroblasts (NAFs), and (B) MCF7 cells treated with recombinant IL1β (rIL1β) for 6 hrs. Average gene expression and standard error of the mean (SEM) from 3 independent experiments are depicted in the bar graphs. (C) Single-cell suspensions from reduction mammoplasty samples were placed in organoid culture and treated with recombinant rIL1β for 8 days and acinar structure formation was examined and photographed. Scale bars represent 400μm. (D) EpCAM and CD49f expression in cells obtained from (C) was examined by flow cytometry. A representative plot is shown and the average percentage of cells in each gate and SEM from 4 independent samples is shown in each quadrant. (E) Total number of viable cells and (F) colony forming cell (CFC) yields are depicted in bar graphs. Average cell numbers and CFC yield with SEM from 4 independent experiments are depicted in the bar graphs. (*P < .05, **P < .005, ***P < .0005 and ****P < .00005)

Supplementary Figure 3 (A-E) related to Figure 3. Fibroblasts from tumour-adjacent tissue (TAT-F) and contralateral breast tissue (CNTB-F) show similar mechanism of IL1β induction in co-cultures. (A) TATFs and CNTB-Fs express similar to NAFs *IL1β* transcript level. (B) *IL1β* transcript expression was measured in TATFs, CNTB-Fs and NAFs co-cultured with MCF7 or T47D cells for 10 days. (C) TATFs, CNTB-Fs and NAFs were treated with conditioned media (CM) obtained from either the co-cultures with MCF7 or T47D cells or breast cancer cells

only cultures for 6 hr and *IL1β* transcript levels were measured. The data is represented as bar graphs with Mean ± SEM from 3 independent experiments. (D) MCF7 and (E) T47D cells were placed in 2 dimensional co-cultures with tumour-adjacent breast tissue-associated fibroblasts (TATFs), or matching contralateral none tumour containing breast fibroblasts (CNTB-Fs) or NAFs for upto 10 days. Expansion of breast cancer cells in these cultures were measured as the number of viable $EpCAM^+$ via flowcytometry at the beginning (input) and on the indicated days (output). All the data is represented either as bar graphs or line graphs with Mean ± SEM from 3 independent experiments. (*P < .05, **P < .005, ***P < .0005 and ****P < .00005)

Supplementary Figure 4 (A-H) related to Figure 4. Normal breast epithelial cells do not produce PDGF-BB in co-cultures with NAFs. (A) IL1R1 expression in NAF, MCF7, and T47D was measured by flow cytometry. (B) IL1β protein expression as measured by cytokine ELISA array in CM from NAFs, $EpCAM^+$ primary normal breast epithelial cells or organoid co-cultures of both cells types. (C) NAFs were treated with CM2 generated from NAFs and $EpCAM^+$ normal epithelial cell interaction for 6 hr and *IL1β* transcript level was measured by qPCR. (D) $EpCAM^+$ normal breast epithelial cells were treated with CM from NAFs for 6hrs and *PDGFB* transcript expression was measured by qPCR. (E) Primary ER+ tumour cells treated with recombinant PDGF-BB for 6hrs, IL1β transcript expression was measured and plotted. (F) NAFs were treated with either recombinant PDGF-BB or vehicle control for 5 days and number of viable (propidium iodide negative) cells was obtained by flow cytometry. (G) NAFs were placed in organoid cultures and treated with escalating doses of SU16f for 48hrs, followed by the addition of conditioned media from primary ER+ breast cancer cells and NAFs (CM2) for 6hrs and IL1β transcript levels were quantified. (H) NAFs were placed in organoid cultures for along with escalating doses of SU16f and cell numbers were counted after 5 days and plotted. All the data are represented as bar graphs with Mean ± standard error of the mean from 3 independent experiments. (*P < .05, **P < .005, ***P < .0005 and ****P < .00005)

Supplementary Figure 5 (A-H) related to Figure 6. TAFs induce proliferation of breast cancer cells through similar fibroblast-initiated mechanisms. (A, B) 2D adherent co-cultures were initiated with $EpCAM^+$ ER^+ breast cancer cell lines (MCF7, T47D) and NAFs and/or TAFs. Total numbers of viable $EpCAM^+$ tumour cells were measured by flow cytometry at the beginning (input) and on indicated days. (C) Total numbers of viable $EpCAM^-$ fibroblasts were also measured at the beginning (input) and after 5 days of cultures. (D) TAFs were treated with either recombinant PDGF-BB or vehicle control for 5 days and numbers of viable cells were measured by flow cytometry. (E) IL1R1 expression in TAF was measured by intracellular flow cytometry. (F) Expression levels of CCL7, IL6 and IL8 were measured in TAF through intracellular flow cytometry and immunofluorescence analysis. Scale bars represent 200μm. All the data are represented as bar graphs with Mean ± SEM from 3 independent experiments (*P < .05, **P < .005, ***P < .0005 and ****P < .00005). (G) Cox fitting and prognostic indicators were used to

generate risk groups from The Cancer Genome Atlas (TCGA) by the SurvExpress online tool. Kaplan-Meier curves of risk group prediction across the TCGA data set are shown. Red line represents high risk and green line represents the low risk group. (H) *PDGFB* and *IL1β* expression in the high risk and low risk group based on prognostic index is shown. (I) Mixture of MCF7 and TAFs were used in an orthotopic mouse tumour model to generate tumours. Mice were either treated with Anakinra (Ana), SU16F (Su), Ana+Su, or vehicle control. Cell-free areas of H&E sections from each tumour was obtained and analyzed as described in the supplementary materials and methods section. Representative areas of analysis from each xenograft section is shown. Areas containing cells (MCF7 and TAFs) are selected (yellow lines) and the ration of cell-free area in each xenograft section was normalized to the total tumour area.

Supplementary Figure 6 (A-F) related to Figure 7. Characterization of ER^+BCC-associated $CD45^+CD31^+$ cells. (A) Tumour-associated fibroblasts (TAFs) or (B) $CD45^+CD31^+$ cells from the primary estrogen receptor positive breast tumours were put into organoid cultures and the number of viable (propidium iodide negative, PI^-) cells after 10 days were obtained by flow cytometry. Line graphs from 3 independent samples are shown. (C) Cytokine ELISA array analysis identified 65 different cytokines secreted by ER^+BCC-associated $CD45^+CD31^+$ cells (also, Table S3). Average from 3 biological replicates and standard error of the mean (SEM) are plotted as bar graphs. (D) Flow cytometric analysis of the ER^+BCC-associated $CD45^+$ cells revealed presence of different immune cells ($CD3^+$ T, $CD19^+$ B, $CD14^+$ Monocytes and $CD56^+$ NK cells) in the tumour microenvironment. Data from 5 independent samples were plotted as dot plots. (E) pi-chart showing different cytokines secreted by different immune cell subsets in the ER^+BCC-associated $CD45^+CD31^+$ cells. (F) MCF7 cells were grown as organoids and treated with different concentrations of tamoxifen (Tam) for 8 days and viable (PI^-) cell numbers were determined by flow cytometry. The data are represented as bar graphs from 3 independent experiments as Mean ± SEM. (*P < .05, **P < .005, ***P < .0005 and ****P < .00005)

Supplementary Figure 1 (A-G) related to Figure 1

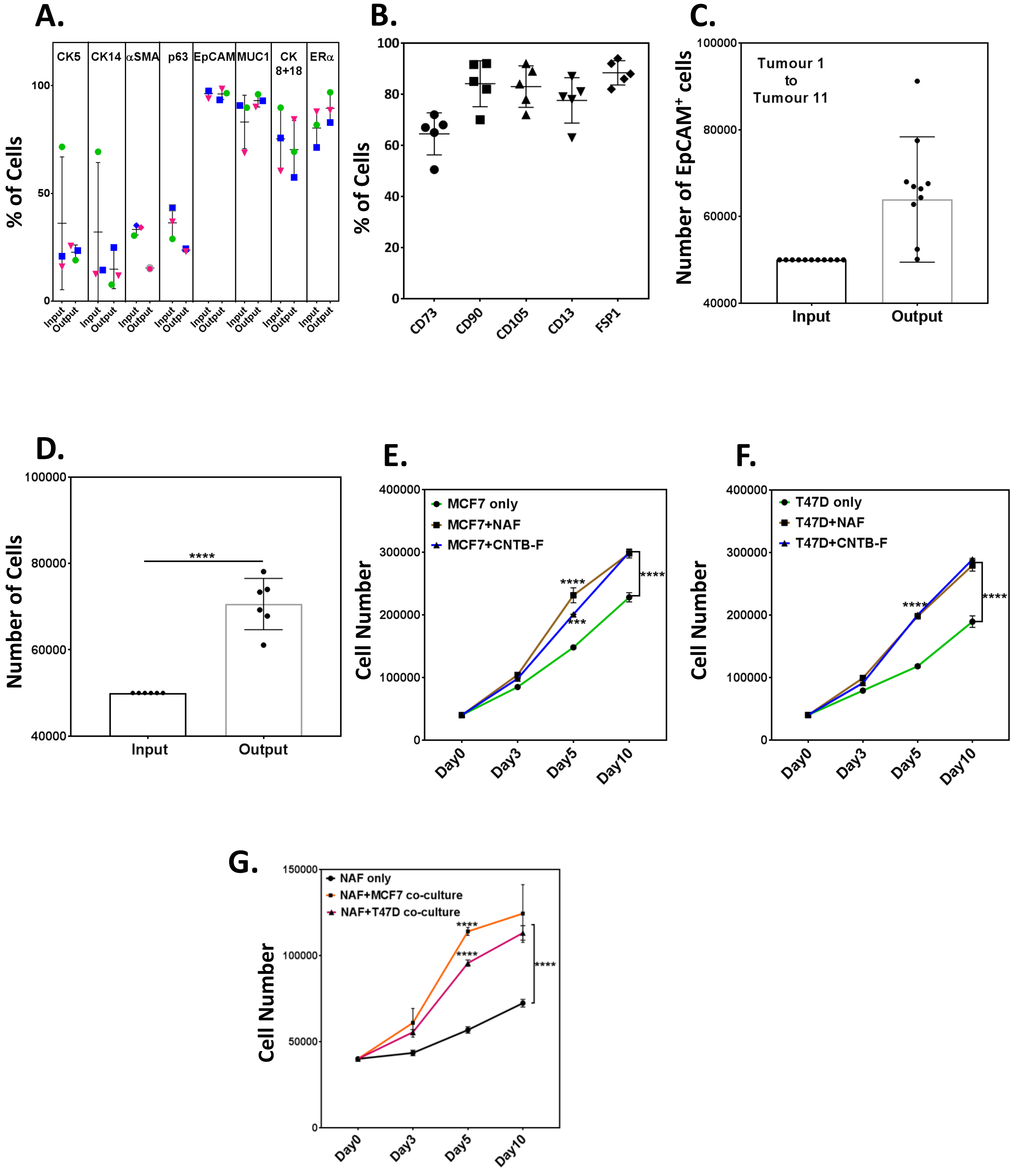

Supplementary Figure 2 (A-F) related to Figure 2

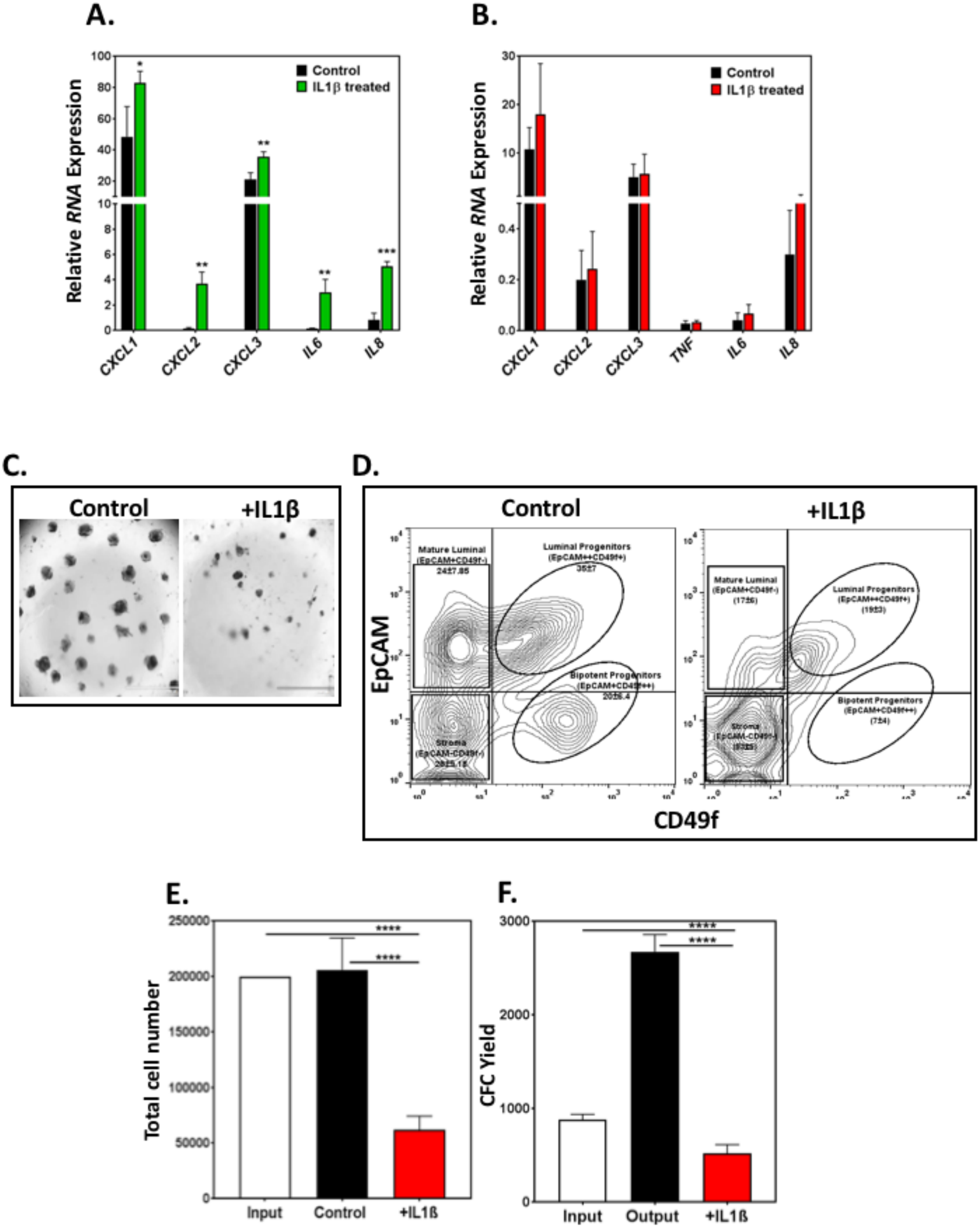

Supplementary Figure 3 (A-E) related to Figure 3

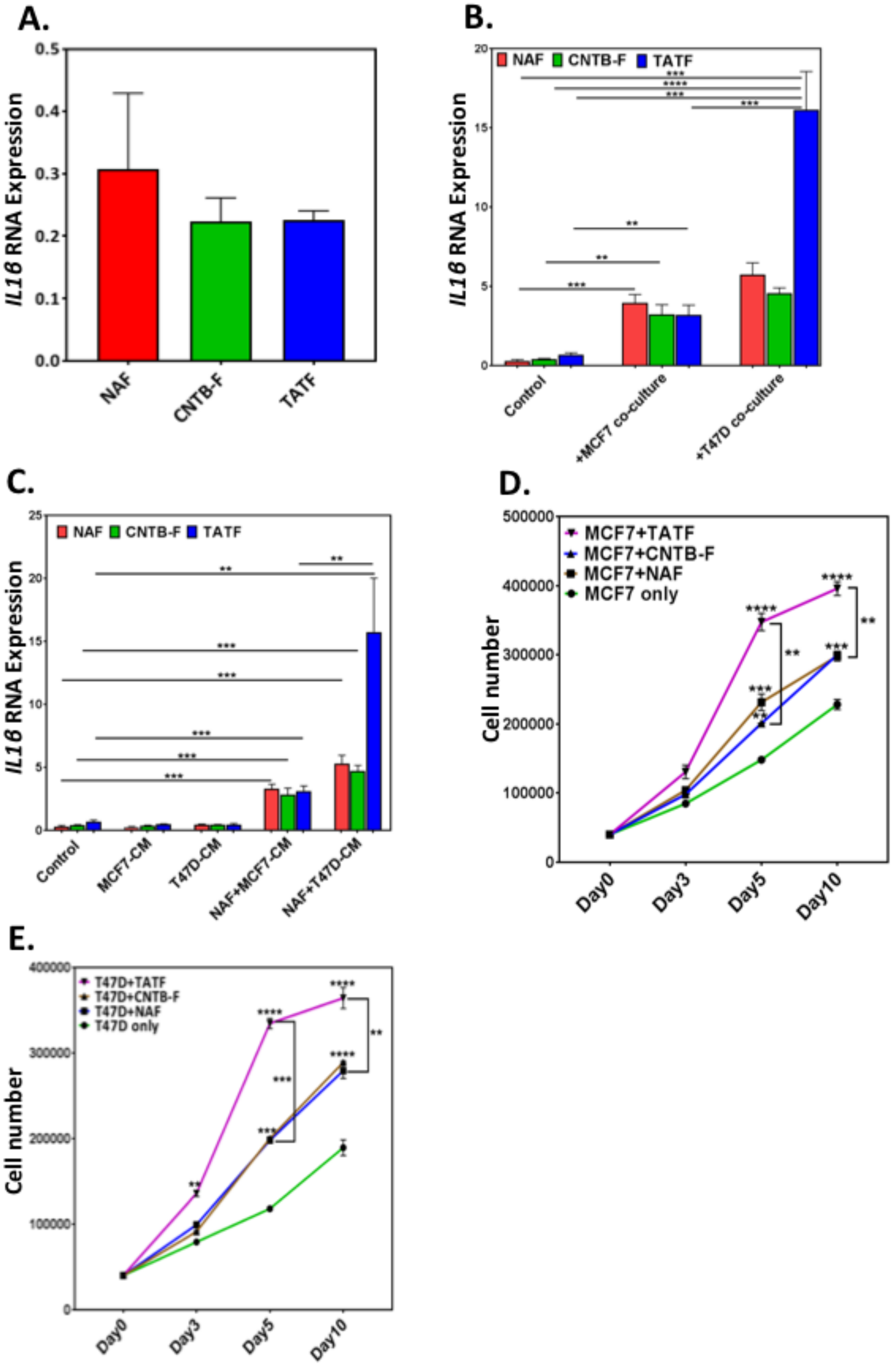

Supplementary Figure 4 (A-H) related to Figure 4

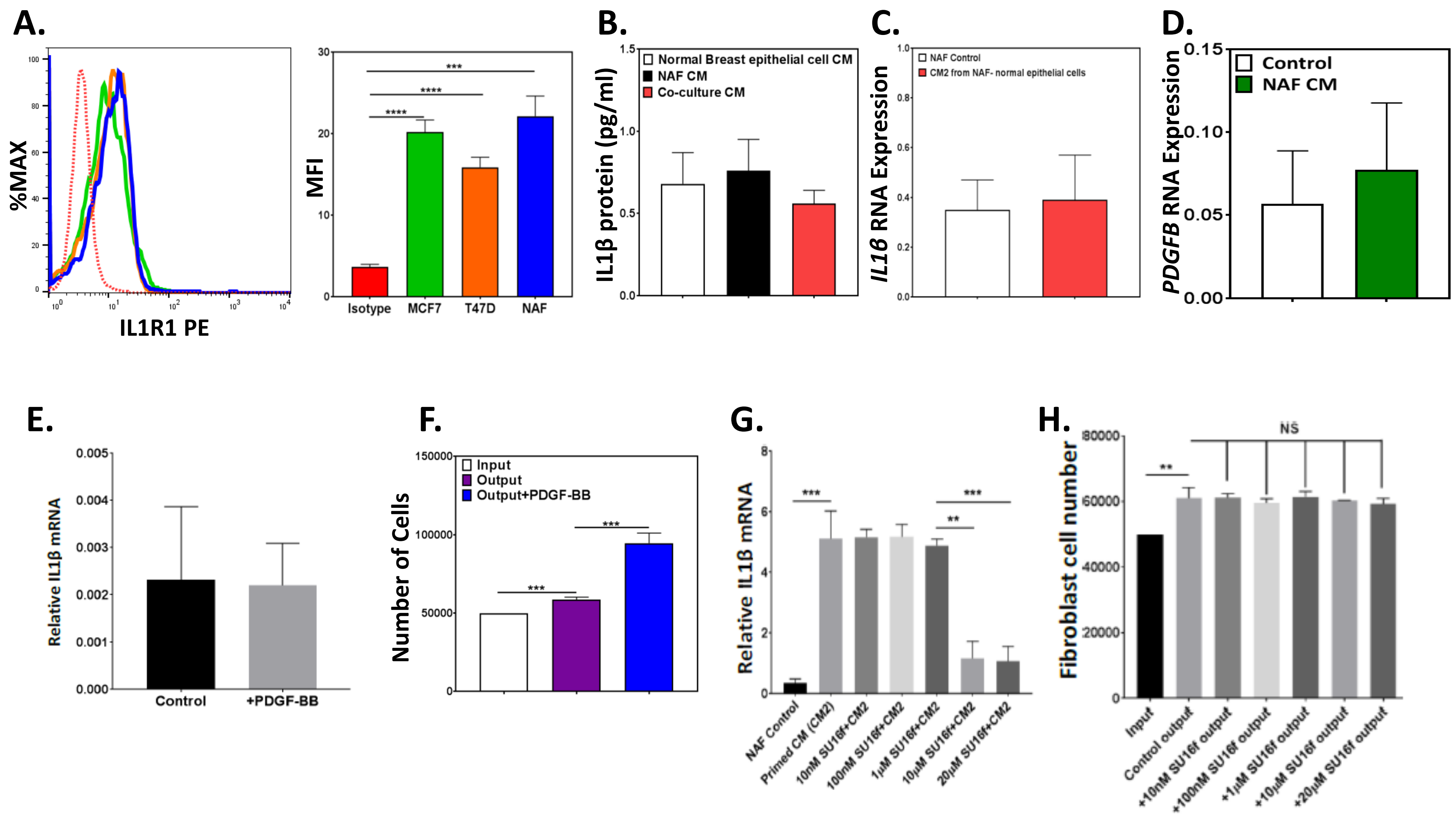

Supplementary Figure 5 (A-H) related to Figure 6.

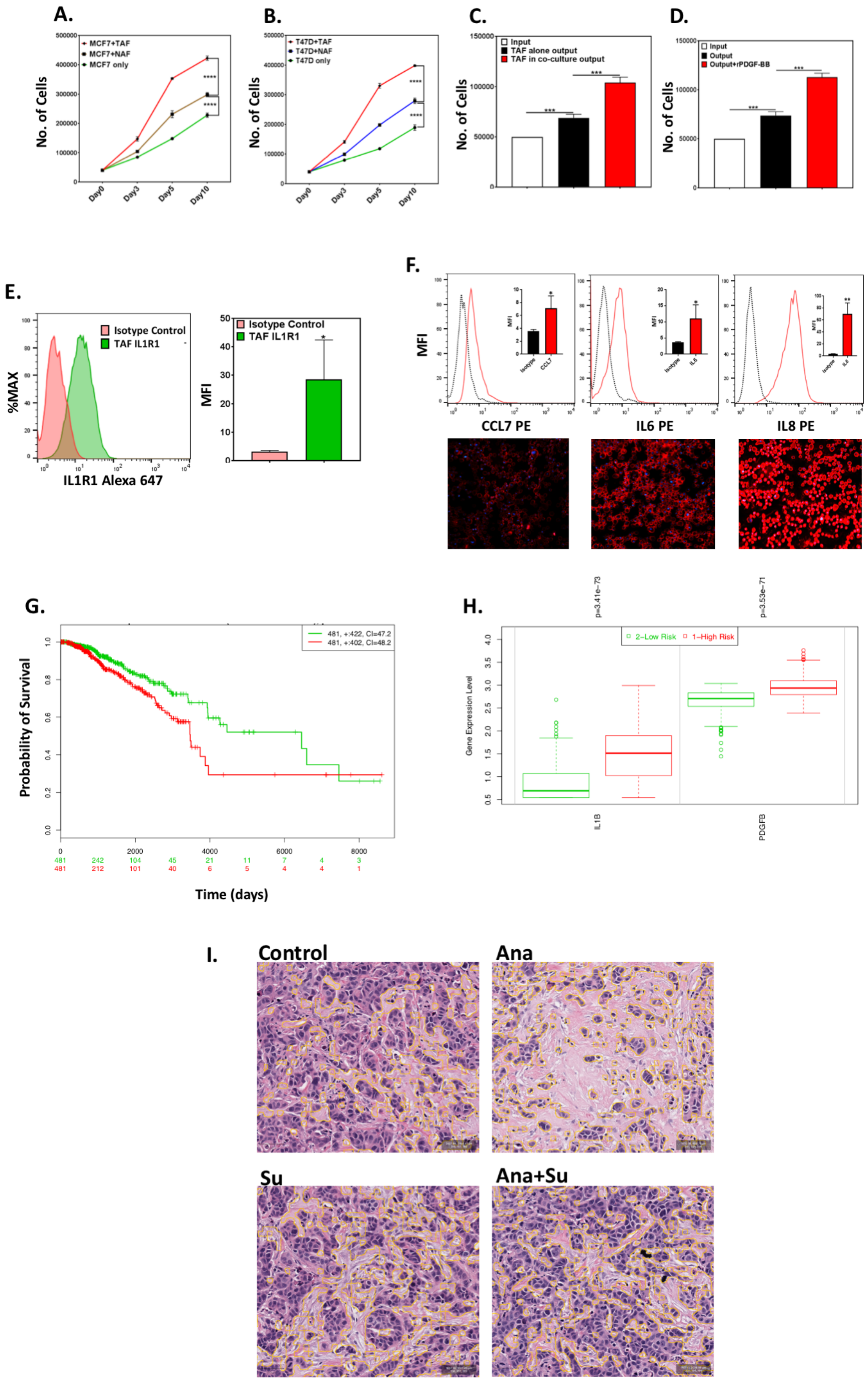

Supplementary Figure 6 (A-F) related to Figure 7

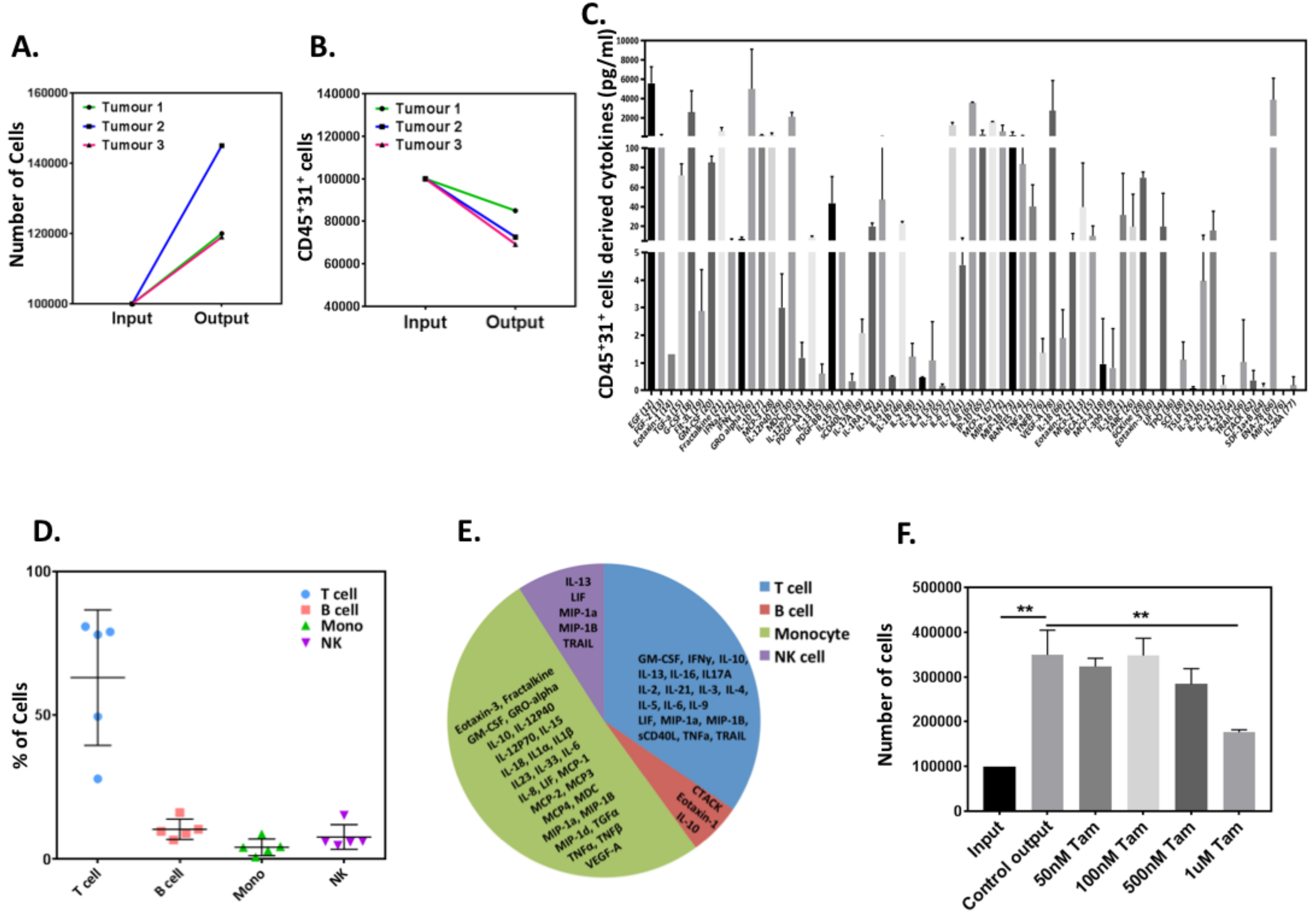

Supplement: Document S1. Transparent Methods and Figures S1–S6 [file mmc1.pdf]
